# Periconception bisphenol and phthalate concentrations in women and men, time to pregnancy, and risk of miscarriage

**DOI:** 10.1016/j.envres.2025.121712

**Published:** 2025-04-29

**Authors:** Sophia M. Blaauwendraad, Aline J. Boxem, Romy Gaillard, Linda G. Kahn, Mathusa Lakuleswaran, Amrit Kaur Sakhi, Eline L. Bekkers, Zixuan Mo, Larry Spadacini, Cathrine Thomsen, Eric AP. Steegers, Annemarie GMGJ. Mulders, Vincent WV. Jaddoe, Leonardo Trasande

**Affiliations:** a The Generation R Study Group, Erasmus University Medical Center, Rotterdam, the Netherlands; b Department of Pediatrics, Erasmus University Medical Center, Rotterdam, the Netherlands; c Departments of Pediatrics and Population Health, New York University School of Medicine, New York, NY, United States; d Department of Food Safety, Norwegian Institute of Public Health, Norway; e Departments of Pediatrics and Environmental Medicine, New York University School of Medicine, New York, NY, United States; f Department of Obstetrics and Gynecology, Erasmus University Medical Center, Rotterdam, the Netherlands; g New York University Wagner School of Public Service, New York City, NY, United States

**Keywords:** Bisphenols, Phthalates, Time to pregnancy, Miscarriage

## Abstract

**Background:**

Exposure to endocrine-disrupting chemicals such as bisphenols and phthalates might lead to adverse fertility and early pregnancy outcomes.

**Methods:**

This study was embedded in the Generation R *Next* Study, a population-based cohort study from preconception onwards. Urinary phthalate and bisphenol concentrations were assessed in the preconception period (938 women), defined as the period in which couples were actively trying to conceive, and early pregnancy (1,366 women and 1,202 men, mean gestational age at sampling 8⋅6 weeks). Time to pregnancy and miscarriage were assessed using questionnaires and ultrasounds. Subfertility was defined as the inability to conceive within 12 months or need for assisted reproductive technologies.

**Findings:**

Higher preconception urinary bisphenol S (BPS) and cyclohexane-1,2-dicarboxylic acid-monocarboxy isooctyl ester (mCOCH) concentrations in women were associated with longer time to pregnancy. Higher preconception mono-[(2-carboxymethyl)hexyl] phthalate, mono-2-ethyl-5-oxohexyl phthalate (mEOHP), mono-(7-carboxy-n-heptyl)phthalate (mCHpP), and mono benzyl phthalate (mBzBP) were associated with shorter time to pregnancy, and higher mono-2-ethyl-5-hydroxyhexyl phthalate (mEHHP), mEOHP, and mBzBP with lower odds of subfertility. In men, higher early pregnancy BPS, mCHpP, mono-4-methyl-7-hydroxyoctyl phthalate, mono-4-methyl-7-oxooctyl phthalate, and mono-ethyl phthalate were associated with shorter time to pregnancy or lower odds of subfertility. Higher preconception or early pregnancy BPS, phthalic acid, and mCHpP in women were associated with lower odds of miscarriage, whereas higher mono-carboxy-isoctyl phthalate, mCOCH, and mono-2-(propyl-6-carboxy-hexyl)-phthalate (cxmPHxP) with higher odds of miscarriage (all p-values <0⋅05).

**Interpretation:**

Preconception and early pregnancy exposure to bisphenols and phthalates may affect couple fertility. Our results should be considered as hypothesis generating and replicated in future studies, possibly including repeated chemical measurements and mixture analysis.

## 1. Introduction

Endocrine-disrupting chemicals such as bisphenols and phthalates are widely used in the production of common consumer products, such as plastic bottles, thermal papers and food can coatings (Koch and Calafat, 2009; Nahar et al., 2015; Woodruff et al., 2011; Ye et al., 2008). An accumulating body of evidence suggest that exposure to these endocrine disrupting chemicals among women of reproductive age affect their pregnancy course and risk of pregnancy complications (Land et al., 2022). In males, exposure to bisphenols and phthalates seems to be associated with abnormal sperm quality (Wang et al., 2016). Bisphenols are xenoestrogens that bind to estrogen receptors alpha and beta, significantly increasing levels of 17-beta-estradiol (Philips et al., 2018a). *In vitro* studies have shown that phthalates may bind to estrogen receptor alpha, exhibiting weak estrogenic effects, and to androgen receptors, exhibiting strong anti-androgenic activity (Philips et al., 2018a). Also, both animal and human studies indicate that bisphenols and phthalates induce oxidative stress, which has been associated with adverse female reproductive outcomes (Ferguson et al., 2014; Watkins et al., 2015; Philips et al., 2017; Yuan et al., 2020; Lu et al., 2018). Only four previous studies, including 164 to 501 couples, assessed the associations of phthalate and bisphenol exposure before pregnancy with time to pregnancy in women and showed inconsistent results (Yeum et al., 2019; Thomsen et al., 2017; Buck Louis et al., 2014; Jukic et al., 2016). These studies had small sample sizes, had maximum follow up times of 6–12 months, and did not include novel bisphenols and phthalates such a bisphenol S, diisononyl phthalates (DINP), and di (isononyl)cyclohexane − 1,2-dicarboxylate (DINCH) (Yeum et al., 2019; Thomsen et al., 2017; Buck Louis et al., 2014; Jukic et al., 2016). Results from previous studies on the associations of bisphenols and phthalates concentrations in women with miscarriage risk were likewise inconsistent (Jukic et al., 2016; Toft et al., 2012; Gao et al., 2017; Mu et al., 2015; Ji et al., 2023). In men, only one previous study among 501 couples reported associations of higher men’s urinary monomethyl (mMP), mono-n-butyl (mBP) and monobenzyl (mBzBP) phthalates with a longer time to pregnancy (Buck Louis et al., 2014).

We hypothesized that exposure to endocrine-disrupting chemicals in the preconception or early pregnancy periods might adversely affect fertility and miscarriage risk. In a population-based prospective cohort study from the preconception period onwards, we assessed the associations of urinary phthalate and bisphenol concentrations before or early in pregnancy with time to pregnancy, subfertility, and risk of miscarriage among 2,304 women and 1,202 men. Our study adds to the existing literature as our cohort is population-based from preconception onwards, includes both women and men, and assessed newer, recently introduced, plasticizer alternatives.

## 2. Methods

### 2.1. Study design

**Fig. 1.**
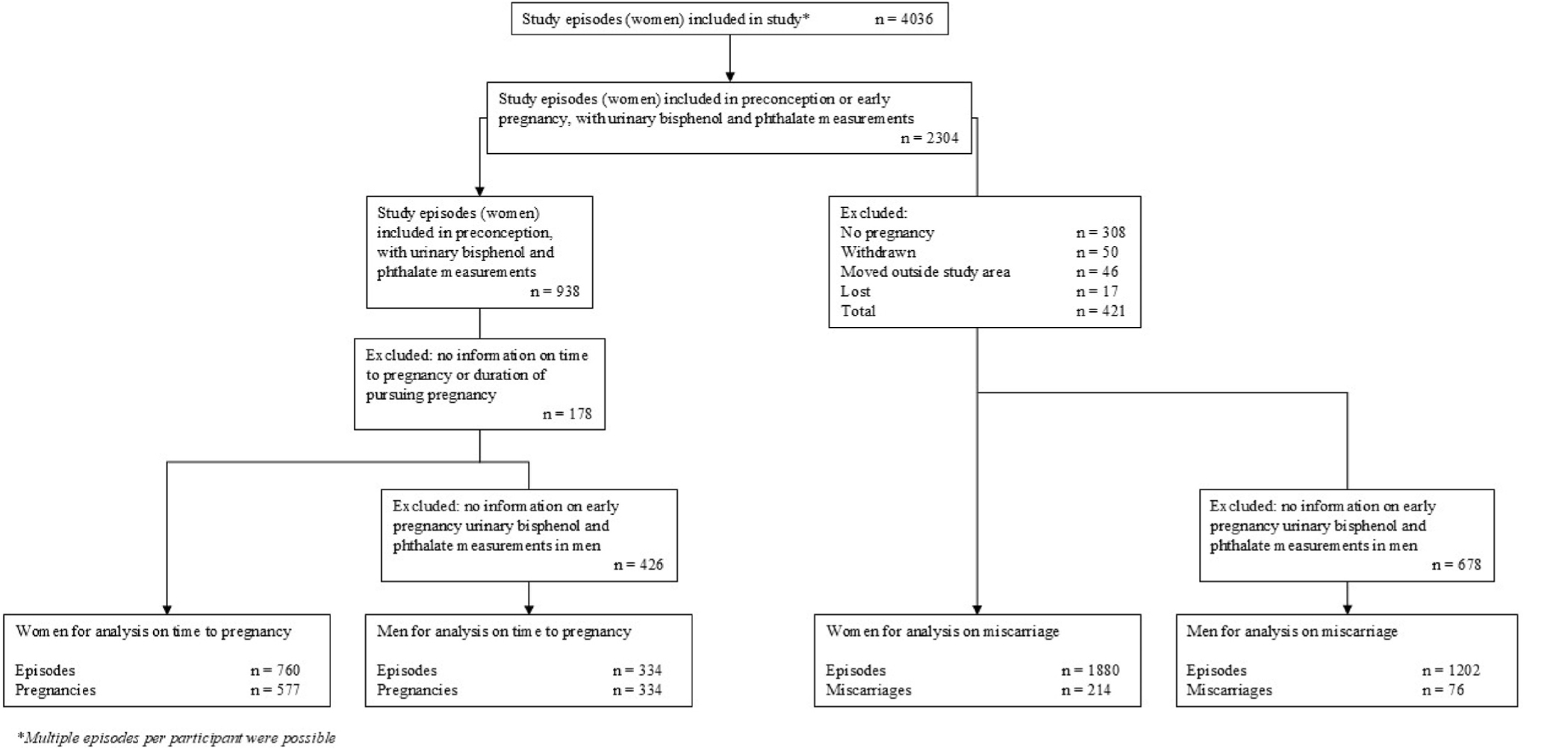
Flowchart of study population.

This study was embedded in the Generation R *Next* Study, a population-based prospective cohort study from the preconception period onwards in the city of Rotterdam, the Netherland, and is part of the Generation R Study Programme (Kooijman et al., 2016). The general aim if this study is to identify preconception and early-pregnancy determinants of fertility, embryonic and childhood outcomes. Women and their partners in the general population were eligible if they were ≥18 years old, living in Rotterdam, and actively trying to conceive or pregnant. Couples were included in preconception period or pregnancy between 2,017 and 2,021, and followed up throughout pregnancy if they conceived before January 2,022. This study aimed to include couples in the preconception period, but allowed inclusion in pregnancy until delivery. Each pregnancy and preconception period prior to that was defined as a study episode, and couples were allowed to participate in multiple episodes. In total, 33⋅2 and 52⋅8 % of all episodes were included in the preconception period or first trimester, respectively. Study approval was obtained by the Medical Ethical Committee of the Erasmus University Medical Centre, Rotterdam (MEC, 2016–589, NL57828.078.16). Written informed consent was obtained from participating women and men. Women could participate more than once for different preconception or pregnancy episodes. In total, there were 4, 036 episodes from 3,604 unique women, leading to 3,577 pregnancies of 3,200 unique women at the end of the study. Urinary bisphenols and phthalates concentrations were assessed in a subgroup of the population with urine samples available. Of the group of 4,036 episodes, 2,304 had information on preconception or early pregnancy urinary bisphenols and phthalates concentrations. Of the 938 episodes with information about preconception urinary bisphenols and phthalates concentrations, 760 episodes (742 unique women) had information on time to pregnancy and subfertility, and 334 (43⋅9 %) of their partners had urinary bisphenol and phthalate measurements in early pregnancy. For the analysis on miscarriage, 1,880 episodes (1,708 unique women) had information on miscarriage, and 1,202 (63⋅9 %) of their partners had early pregnancy urinary bisphenols and phthalates measurements (Flowchart shown in Fig. 1).

### 2.2. Urinary bisphenol and phthalate assessments

Concentrations of eight bisphenols and 30 phthalates were quantified in spot urine samples obtained from each woman at enrollment in the preconception period and in early pregnancy, and from each man in early pregnancy. In women that had multiple samples in the preconception period, we used the most recent sample. Analyses were performed at the Wadsworth Center, New York State Department of Health, Albany, which later moved to the New York University School of Medicine (NYU), New York, USA, and at the Norwegian Institute of Public Health (NIPH), Oslo, Norway, under close collaboration and through comparable methods (Text S1). Details of bisphenol, phthalate, and creatinine concentration assessment via liquid chromatography-tandem mass spectrometry have been described earlier (Philips et al., 2018a; Sabaredzovic et al., 2015; Sakhi et al., 2018). Values below the limit of detection (LOD) were imputed as the LOD divided by the square root of 2 (Hornung and Reed, 1990). Chemicals that were detected in more than 20 percent of samples were assessed individually and included in grouping. We used the cut-off of 20 percent because this cut-off enabled us to include the maximum number of participants in the analysis with adequate variability in the bisphenol and phthalate data to detect associations. This approach is in line with previous studies in the field (Blaauwendraad et al.). For all chemicals, the descriptive statistics and percentage below the limit of detection in women and men are shown in Table S1 and S2. Phthalate metabolites were grouped according to their molecular weight and parent compounds (details Text S2). We included low molecular weight phthalates [LMW], and high molecular weight [HMW] phthalates of the subgroups di-2-ethylhexyl phthalate [DEHP], di-n-octyl phthalate [DNOP], DINCH, DINP, and diisodcyl phthalate [DiDP]). We grouped eligible bisphenols into a total bisphenols group. Those were bisphenol A and bisphenol S. Phthalic acid [PA] was analyzed as a proxy for total phthalate exposure, as most phthalates are metabolized to phthalate monoesters in the human body. In women that were included in the miscarriage analysis, preconception chemicals concentrations were substituted with early pregnancy chemicals concentration when preconception urine was not available. We prioritized preconception measurements, as we were most interested in the effect of preconception exposure to chemicals. To account for urinary dilution, we adjusted for urinary creatinine concentrations in all our models (O’Brien et al., 2016). To account for potential variability in values between laboratories, we standardized the bisphenol and phthalate concentrations by dividing them by their laboratory-specific interquartile range (IQR). Hereafter, urinary bisphenol and phthalate concentrations were log2 transformed to account for right skewedness.

### 2.3. Time to pregnancy and miscarriage

Time to pregnancy and mode of conception were assessed through questionnaires in preconception period and early pregnancy. We used questions regarding the date at which women started trying to conceive and refrained from using contraceptives (start date actively pursuing pregnancy) and the date of assisted reproductive technology, including intrauterine insemination (IUI), ovulation induction, in vitro fertilization (IVF), intracytoplasmic sperm injection (ICSI). These questions were confirmed by their obstetric care giver, at each yearly preconception visit, and at the early pregnancy visit. Pregnancy status of the women was self-assessed by urinary pregnancy tests and confirmed by the caregiver through ultrasounds. The first day of the last menstrual period was obtained from obstetric caregiver. In women who did conceive, time to pregnancy was calculated from the start of actively pursuing pregnancy and their first day of last menstrual period. In women who used assisted reproductive technologies we added 12 months to their time to pregnancy, as fertility treatments usually start after one year of not conceiving (Infertility Workup for the Women, 2019). In women that did not conceive, the duration of actively pursuing pregnancy was calculated from the start of actively pursuing pregnancy and the last day of study participation or the study end date. Fecundability was defined as the probability of conceiving within one month. Time to pregnancy was categorized in two groups: 0–12 months (fertile) and >12 months (subfertile). Women with a duration of child wish without pregnancy for >12 months or who underwent assisted reproductive technology were included in the subfertile group. Miscarriage was defined as a loss of normally implanted pregnancy before 22 weeks of gestation (Pregnancy et al., 2020). Date of miscarriage was obtained from the participants’ obstetric caregiver. Gestational age at miscarriage was based on either the first day of the last menstrual period, due date based on ultrasound, date of IUI, or embryo implant day minus 14 days in case of IVF or ICSI.

### 2.4. Covariates

Information on age, ethnicity and highest education level was obtained through questionnaires at enrolment. Ethnicity was assessed by the country of birth of the participant and her/his parents. If one of the participants’ parents was born abroad, participants were classified as of non-Dutch ethnic origin (Statistics CBf, 2003; Troe et al., 2007). If both parents were born abroad, the birth country of the mother defined the participants’ ethnicity. Information on smoking, alcohol use, parity and history of previous miscarriage was assessed through questionnaires filled in during preconception and/or early pregnancy. Height and weight were measured at the time of enrollment without shoes and body mass index (BMI) was calculated.

### 2.5. Statistical analysis

First, we examined distributions of population characteristics. We explored the characteristics of fertile and subfertile couples, and of the couples with and without miscarriage. We performed a non-response analysis comparing our study sample to all women and men with bisphenol and phthalate measurements. We explored Spearman’s correlation coefficients of the pooled preconception and early pregnancy chemical concentrations in women and men, and between women and men of the same couple. We explored Spearman’s correlation coefficients of the pooled preconception and early pregnancy chemical concentrations in women and men, and between women and men of the same couple (Hinkle et al., 2003). Second, we assessed the associations of individual and grouped preconception chemicals in women and men and fecundability using Cox proportional hazard models (described in detail in Text S3). The survival outcome was conception or no conception. The time variable was based on time to pregnancy, or the duration of actively pursuing pregnancy among couples without conception. The resulting HR from the Cox model is the fecundability ratio, representing the odds of conceiving per menstrual cycle. Third, we assessed the associations of the chemicals in women and men with the odds of subfertility using logistic regression models. Fourth, we examined the associations of periconception chemicals in women and men with the odds of miscarriage using logistic regression models. In all regression steps, basic models were adjusted for urinary creatinine concentrations and laboratory, and adjusted models were additionally controlled for sociodemographic and lifestyle factors. Potential confounders were selected from previous literature and defined using a Directed Acyclic Graph (Fig. S1). The final adjustment set for women included age, parity, educational level, ethnicity, alcohol use, smoking, and folic acid supplementation. The final adjustment set for men included age, body mass index, and ethnicity, and women’s chemicals concentrations, parity, education, alcohol use, smoking, and folic acid supplementation use. As a sensitivity analysis, we repeated our adjusted analysis in women who conceived spontaneously only. Missing values of covariates and chemical concentrations were imputed using multiple imputation by the fully conditional specification method, and pooled results from 25 imputed datasets were reported. From a hypothesis generating perspective, we considered nominal p-values <0⋅05. To account for multiple hypothesis testing, we considered false discovery rate (FDR)-adjusted p-values of <0⋅05 using the Benjamini-Hochberg method. Analyses were performed using R Statistical Software version 4.3.0.

## 3. Results

### 3.1. Characteristics of study population

Table 1 describes the general characteristics of our study population. Among the 760 women were included in the time to pregnancy analysis, the median time to pregnancy was 7⋅4 months (95 % range 0⋅0 to 84⋅9) and 14⋅2 % conceived through assisted reproduction. Among the 334 men included in time to pregnancy analysis, the median time to pregnancy was 6⋅0 months (95 % range 0⋅0 to 66⋅4) and 15⋅9 % conceived through assisted reproduction. The prevalence of miscarriage was 11⋅4 % among 1880 women included in miscarriage analysis, and 6⋅4 % among 1202 men included in miscarriage analysis. Of the 334 men included in analysis for time to pregnancy, the median gestational age of urinary sampling for chemical measurement was 8⋅1 weeks (95 % CI 6⋅1, 31⋅1). Of the 1,880 women included in analysis for miscarriage, 599 had both preconception and early pregnancy urinary samples for chemical measurements. In those women, we used the preconception sample for our analysis. 1281 had early pregnancy samples only (median gestational age 8⋅6 weeks (95 % CI 6⋅0, 13⋅1). Of the 1,202 men included in miscarriage analysis, the median gestational age of sampling was 8⋅6 weeks (95 % CI 6⋅0, 31⋅1 weeks)). As compared to all women and men with bisphenol and phthalate measurement, women included in the study were slightly older, women used more alcohol in 3 months prior to pregnancy, more often conceived and less often had miscarriages (Table S3). Table S4 shows that compared to the fertile couples, subfertile coupler were older, women were less often Dutch, smoked more often and drank alcohol in pregnancy less often. As compared to couples without miscarriage, couples with miscarriage were older (Table S5). Correlations between the individual chemicals of the same exposure groups were low or very low among women and men (Fig. S2). In general correlations between the individual chemicals within the same high-molecular-weight subgroup were high.

### 3.2. Time to pregnancy and subfertility

**Fig. 2.**
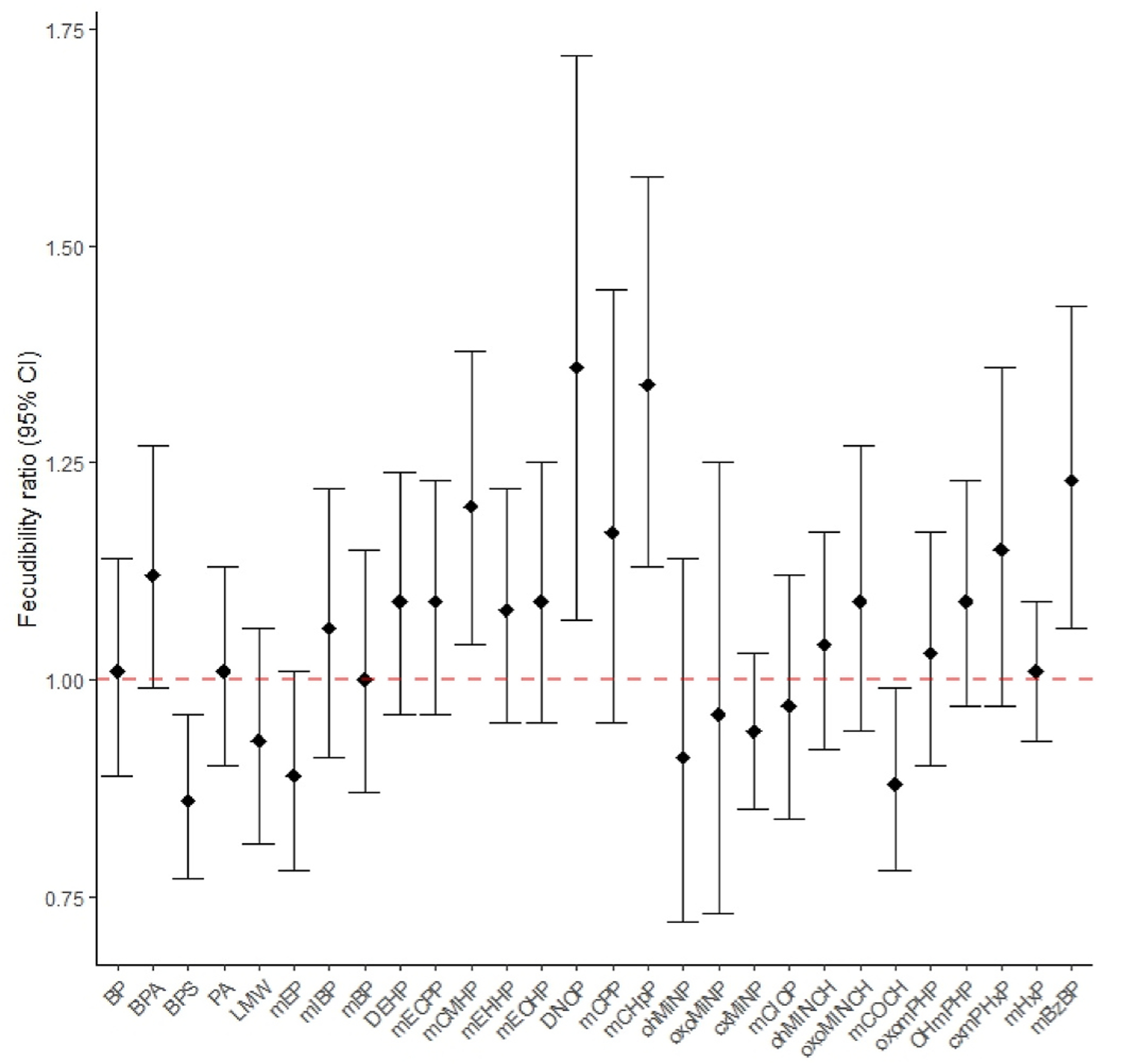
Fecundability ratio for individual and grouped preconception urinary bisphenol and phthalate concentrations in women. Values represent change in fecundability per interquartile range increase in urinary exposure concentration. Cox proportional hazards models are adjusted for age, education, ethnicity, parity, smoking, alcohol use, folic acid supplement use, body mass index, laboratory of analysis, and maternal urinary creatinine concentration. Corresponding data are presented in Table S4.

Higher preconception urinary concentrations of bisphenol (BPS) and cyclohexane-1,2-dicarboxylic acid-monocarboxy isooctyl ester (mCOCH) in women were associated a lower fecundability, reflecting a longer time to pregnancy (FR BPS 0⋅86 [95 % confidence interval (CI) 0⋅79, 0⋅96] and mCOCH 0⋅88 [95 % CI 0⋅78, 0⋅99] per IQR increase in chemical) (Fig. 2, Table S6–S7). In contrast, higher preconception concentrations of mono-[(2-carboxymethyl)hexyl]phthalate (mCMHP), mono-(7-carboxy-n-heptyl)phthalate (mCHpP), and mBzBP in women were associated with higher fecundability, reflecting a shorter time to pregnancy (FR mCMHP 1⋅20 [95 % CI 1⋅04, 1⋅38], mCHpP 1⋅34 [95 % CI 1⋅13, 1⋅58], and mBzBP 1⋅23 [95 % CI 1⋅06, 1⋅43]). In men, higher early pregnancy urinary concentrations of mCHpP, ohMiNP, and oxoMiNP were associated with higher fecundability, reflecting a shorter time to pregnancy (FR mCHpP 1⋅32 [95 % CI 1⋅09, 1⋅60], ohMiNP 1⋅77 [95 % CI 1⋅15, 2⋅76] and 1⋅71 [95 % CI 1⋅09, 2⋅69] per increase in chemical) (Table S8). In women and men, no associations of the bisphenol or phthalate groups with fecundability were present. Only the association of higher preconception mCHpP with higher fecundability in women remained significant after FDR correction.

**Fig. 3.**
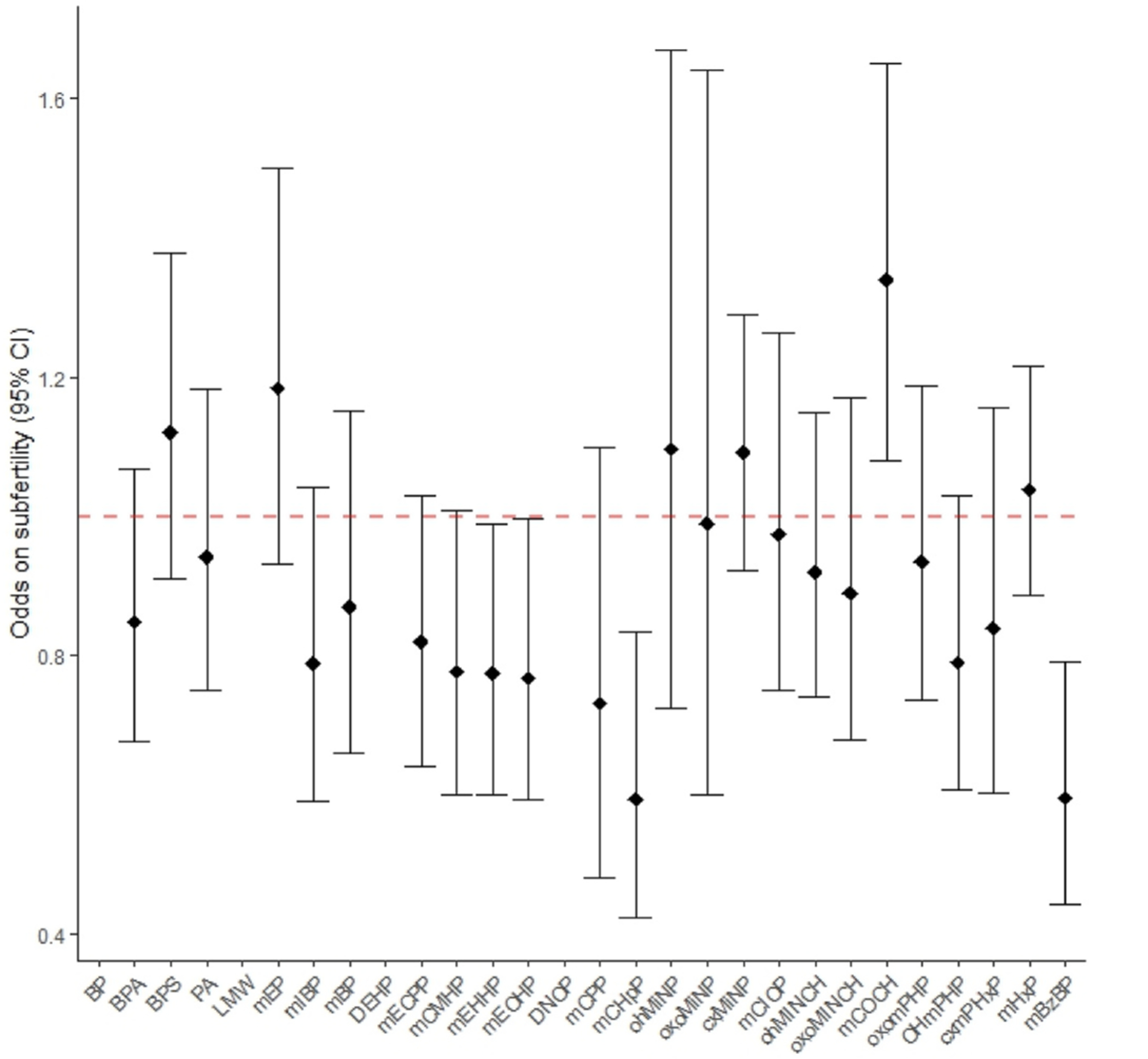
Associations of individual and grouped preconception urinary bisphenol and phthalate concentrations with subfertility in women. Values represent odds of subfertility (odds ratio (OR), 95 % confidence interval (CI)) per interquartile range increase in urinary exposure concentration. Logistic regression models are adjusted for age, education, ethnicity, parity, smoking, alcohol use, folic acid supplement use, body mass index, laboratory of analysis, and maternal urinary creatinine concentration. Corresponding data are presented in Table S7.

Higher preconception mono-2-ethyl-5-hydroxyhexyl phthalate (mEHHP), mono-2-ethyl-5-oxohexyl phthalate (mEOHP), mCHpP and mBzBP in women were associated with lower odds of subfertility (OR mEHHP 0⋅77 [95 % CI 0⋅60, 0⋅99], mEOHP 0⋅77 [95 % CI 0⋅59, 1⋅00], mCHpP 0⋅59 [95 CI 0⋅42, 0⋅83], and mBzBP 0⋅59 [95 % CI 0⋅44, 0⋅79]) (Fig. 3, Table S9–S10). Higher early pregnancy BPS in men was associated with higher odds of subfertility and higher mEP with lower odds of subfertility (OR BPS 1⋅37 [95 % CI 1⋅00, 0⋅88], and mEP 0⋅63 [95 % CI 0⋅43, 0⋅91]) (Table S11). We did not observe associations of the bisphenol or phthalate groups in women and men with subfertility. Of the significant associations, these of higher mCHpP and mBzBP in women with lower odds of subfertility remained significant after FDR correction.

### 3.3. Miscarriage

**Fig. 4.**
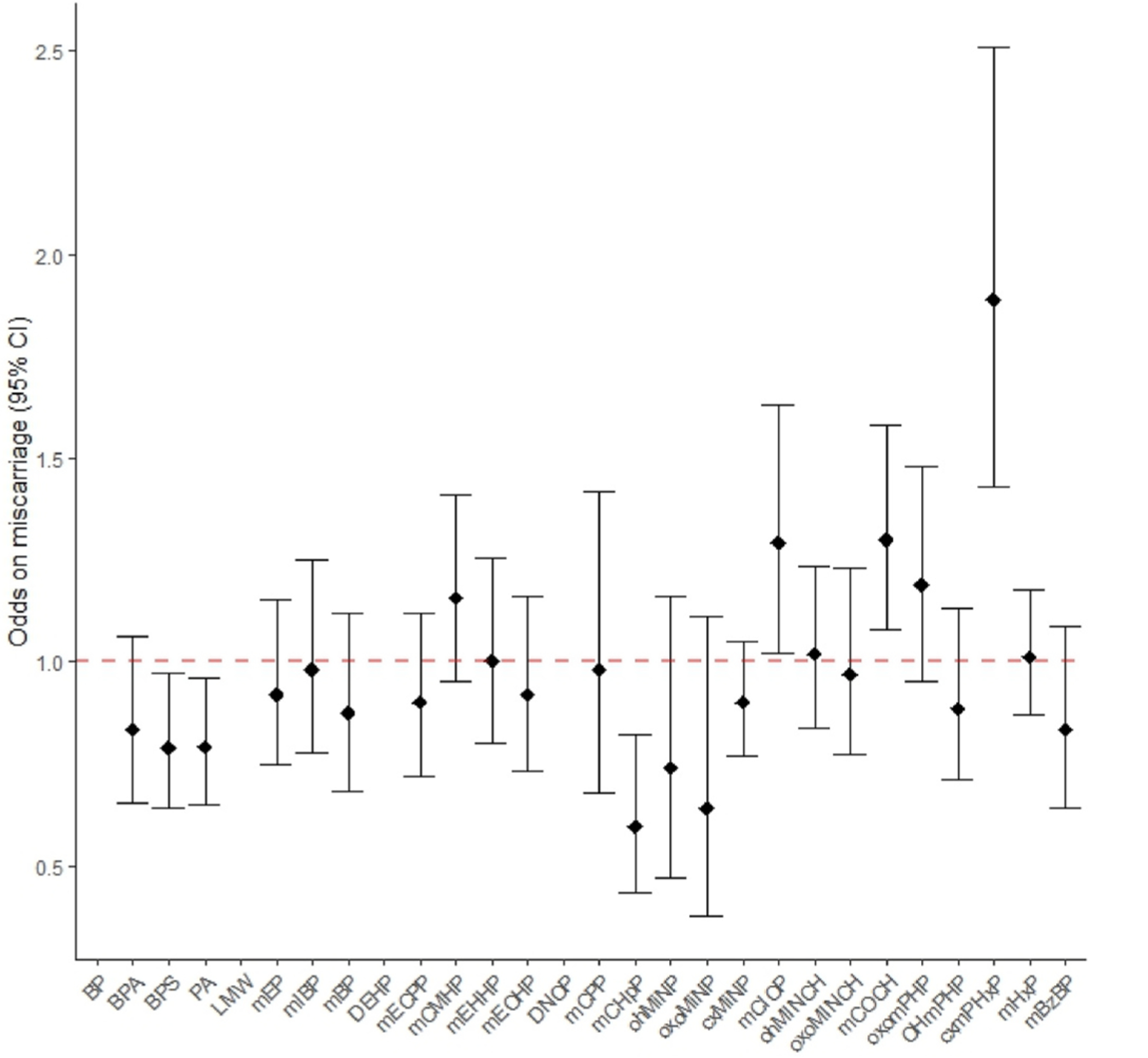
Associations of individual and grouped periconception urinary bisphenol and phthalate concentrations with miscarriage in women. Values represent odds of miscarriage (odds ratio (OR), 95 % confidence interval (CI)) per interquartile range increase in urinary exposure concentration. Logistic regression models are adjusted for age, education, ethnicity, parity, smoking, alcohol use, folic acid supplement use, body mass index, laboratory of analysis, and maternal urinary creatinine concentration. Corresponding data are presented in Table S10.

Higher periconception BPS, PA, and mCHpP concentrations in women were associated with lower odds of miscarriage (OR BPS 0⋅79 [95 % CI 0⋅64, 0⋅97], PA 0⋅79 [95 % CI 0⋅65, 0⋅96], and mCHpP 0⋅59 [95 CI 0⋅43, 0⋅82]) (Fig. 4, Tables S12–S13). Higher mono-carboxy-isoctyl phthalate (mCIOP), cyclohexane-1,2-dicarboxylic acid-monocarboxy isooctyl ester (mCOCH), and mono-2-(propyl-6-carboxy-hexyl)-phthalate (cxmPHxP) were associated with higher odds of miscarriage (OR mCIOP 1⋅29 [95 % CI 1⋅03, 1⋅63], mCOCH 1⋅30 [95 % CI 1⋅08, 1⋅58], and cxmPHxP 1⋅89 [95 CI 1⋅43, 2⋅51]). We did not observe associations of the bisphenol or phthalate groups with miscarriage. The associations of mCHpP and cxmPHxP with miscarriage remained significant after FDR correction. No associations of early pregnancy chemicals concentrations in men with miscarriage were present (Table S14).

### 3.4. Sensitivity analysis

In general, results among women who conceived spontaneously were in the similar direction compared to those among the whole study sample (Table S15–S17).

## 4. Discussion

In this population-based prospective cohort study from the preconception period onwards, we observed associations of higher preconception urinary concentrations of BPS and mCOCH in women with a longer time to pregnancy, and of several HMW phthalates with shorter time to pregnancy and lower odds of subfertility. Higher periconception BPS, PA, and mCHpP concentrations in women were associated with lower odds of miscarriage, whereas higher mCIOP, mCOCH, and cxmPHxP were associated with higher odds of miscarriage. In men, higher early pregnancy mCHpP, ohMiNP, and oxoMiNP were associated with a shorter time to pregnancy, and higher BPS and mEP were associated with lower odds of subfertility.

### 4.1. Interpretation of main findings

In women undergoing fertility treatment, phthalate exposure, in particular DEHP exposure, seems to be associated with poor IVF and pregnancy outcomes, such as lower total and mature oocyte yield, fewer clinical pregnancies, and fewer live births (Land et al., 2022). However, among the general population, studies on the association of bisphenol and phthalate exposure in women with fecundability are less conclusive. Four studies assessed associations of female phthalate and bisphenol exposure in the preconception period with time to pregnancy (Yeum et al., 2019; Thomsen et al., 2017; Buck Louis et al., 2014; Jukic et al., 2016). A United States (US) study measuring urinary BPA concentrations in 164 women reported no associations with time to pregnancy over a follow-up period of 12 months (Yeum et al., 2019). Two other US studies, among 221 women followed for 6 months and 501 women followed for 1 year, reported no associations of BPA or several LMW and HMW phthalates with time to pregnancy (Buck Louis et al., 2014; Jukic et al., 2016). Among 229 Danish women, higher mEP was associated with longer time to pregnancy after a maximum follow-up time of 6 menstrual cycles, but there were no associations of mBP, mBzBP, or mEHP (Thomsen et al., 2017). Three studies measured early pregnancy bisphenols and phthalates in women as a proxy for preconception exposure, of which only one reported an association of higher DEHP with a shorter time to pregnancy (Vélez et al., 2015; Specht et al., 2015; Philips et al., 2018b). In men, one previous study among 501 couples reported associations of higher urinary mMP, mBP, and mBzBP with a longer time to pregnancy (Wang et al., 2016; Buck Louis et al., 2014).

The current analysis is the first to study the association of chemicals that have been recently introduced to the industry as replacement for more conventional chemicals with fecundability. Examples are BPS, DINP and DINCH metabolites such as mCOCH. In women, we observed lower odds of conceiving per menstrual cycle after higher preconception exposure to BPS and mCOCH. For the more conventional phthalates, we detected associations of several HMW metabolites, in particular mBzBP, with lower odds of subfertility, which is not in line with previous studies that reported associations of higher exposure to mBzBP with a longer time to pregnnacy. In men, higher early pregnancy BPS, ohMiNP and oxoMiNP, which have not been previously investigated, were associated with a shorter time to pregnancy or lower odds of subfertility. Contrary to the previous study on male exposure that reported associations of higher exposure to LMW phthalates with a longer time to pregnancy, higher mEP in men was associated with lower odds of subfertility. Differences might be explained by our larger sample size and longer follow-up time. Also, studies measuring chemicals in women in early pregnancy are prone to selection bias, as they only include couples that did conceive, and pregnancy might influence chemicals concentrations. Our study, together with results from previous studies, suggest an effect of preconception exposure to bisphenols and phthalates on couple fecundability in the general population, in particular BPS, DINCH and HMW phthalates in women and DINP and LMW phthalates in men.

Several case-control studies have reported higher urinary HMW concentrations in women who experienced miscarriage as compared to controls (Mu et al., 2015; Ji et al., 2023; Yi et al., 2016). Prospective studies of exposure to bisphenols and phthalates and subsequent miscarriage are less conclusive. One study among 221 US women measured bisphenols and phthalates in the preconception period and followed women for 6 months, reporting an association between higher urinary mEOHP and lower risk of pregnancy loss before 6 weeks of pregnancy (Jukic et al., 2016). By contrast, higher early pregnancy BPA in 115 women has been associated with a higher risk of miscarriage (Lathi et al., 2014). Among 128 Danish women with a follow-up time of 6 months, higher periconception mEHP, but not mEP, mBP, and mBzP, was associated with pregnancy loss in the first ten days after conception, but not with later pregnancy loss (Toft et al., 2012). However, this study created selection bias by excluding 77 women who conceived in the first menstrual cycle. A study among 3220 Chinese women reported increased risks of clinical pregnancy loss in women after higher early pregnancy exposure to mEP, mBP, mEOHP, and mEHHP (Gao et al., 2017). In the current study, we observed associations of higher periconception BPS, PA, and mCHpP in women with lower odds of miscarriage, and higher mCIOP, mCOCH, and cxmPHxP with higher odds of miscarriage. Thus several phthalates and bisphenols have shown to be associated with risk of miscarriage, of which some are again the newly emerging DINP and DINCH metabolites. The current study is the first and largest to include preconception and male chemicals concentrations in relation to miscarriage up to 22 weeks of gestation, and replication of our results is warranted.

Several mechanisms have been proposed through which bisphenols and phthalates might negatively impact fertility. Bisphenols and phthalates have shown to increase oxidative stress and alterations in DNA methylation, amongst others by binding to peroxisome proliferator-activated receptor (PPAR) gamma. Oxidative stress and alterations in DNA methylation have been associated with reproductive disorders, and with recurrent miscarriage (Ferguson et al., 2012; Ferguson Kelly et al., 2015; Gupta et al., 2007; Laven, 2022; Menezo et al., 2016; Quenby et al., 2021; Panagiotou et al., 2021). This is supported by in vitro and *in vivo* studies of that report increased oxidative stress, reduced follicle growth and increased atresia after phthalate exposure. Finally, endocrine-disrupting chemicals might interfere with the hypothalamic-pituitary-gonadal axis in both men and women, potentially affecting sperm production or the menstrual cycle, and reducing fecundity. This theory supported by epidemiologic evidence linking BPA in particular with polycystic ovarian syndrome and potentially explaining our reported association of BPS with longer time to pregnancy (Akgül et al., 2019; Barrett and Sobolewski, 2014; Kahn et al., 2020). Also, classic EDCs have been associated with abnormal sperm quality (Wang et al., 2016). Although DINCH and DiNP have been introduced as saver phthalate alternatives for conventional phthalates, their metabolites have shown to exert comparable effect on estrogen, androgen and PPAR receptors in vitro (Yang et al., 2021; Engel et al., 2018).

Our results need replication in future studies, especially those that include populations with higher exposure to newly introduced endocrine-disrupting chemicals. Also, investigation of long term follow-up outcomes such as birth outcomes might contribute to further elucidating the transgenerational effect of bisphenol and phthalate exposure.

### 4.2. Methodological considerations

The current paper benefited from a large, unselected study population, prospective data collection from the preconception period onward, and the availability of a broad range of covariates in women and men. We are one of the few cohorts to have chemical measures in both women and men. Also, as compared to previous studies, our follow up time was long. Also, we included many bisphenols and phthalates, including relatively novel and less researched chemicals such as BPS and DINCH metabolites. As this study is among the first measuring chemical concentrations in the preconception period and including novel chemicals, our aim was to analyze associations of individual chemical from a hypothesis generating perspective. To mimic real-life chemical exposure and account for potential non-additive interactions of the chemicals, future studies should include mixture analysis. Overall, correlations between the chemicals were generally low, except among those within the same high-molecular-weight subgroup, likely reflecting shared sources of exposure. As a result, the risk of co-pollutant confounding is considered low, though it cannot be entirely excluded. We replaced phthalate and bisphenol concentrations below LOD with the LOD divided by the square root of 2. This method has shown to provide an accurate estimation of the mean and standard deviation. Overall, the percentages of metabolites below LOD were reasonable (<50 %), except for mMP in women and men (74.6 % and 79.6 % below LOD), mCMHP in men (69.4 % below LOD), mCHpP in women (67.4 below LOD), and mHxP in women and men (75.3 % and 65.2 % below LOD) in women. This might have reduced variability in the exposure and therefore the ability to detect associations. For the miscarriage analysis, we substituted the preconception chemical measurements with early pregnancy measurements. Although previous studies have shown that spot urinary samples of chemicals might reasonably reflect exposure upon three months prior, life-style adaptations due to pregnancy might have biased these concentrations. We had no information on temporal pauses in actively pursuing pregnancy, which might have occurred in couples due to personal circumstances. We assumed this includes a small number of cases and we yearly confirmed the date of active pursuing pregnancy, but cannot exclude misclassification bias of the outcome for these specific couples. Also, the occurrence of subclinical miscarriages that were not reported by participants may have led to misclassification bias of the outcome miscarriage. Bisphenols and phthalates are non-persistent and have short biological half-lives. In addition, in women, we substituted preconception samples of the chemicals with early pregnancy samples, to increase sample size and power, and to avoid selection bias due to differences in baseline characteristics in women that enrolled before and after onset of pregnancy. However, couples might change lifestyle behaviors throughout the course of trying to conceive or as soon as they are pregnant. Thus, this temporal variability might have led to misclassification bias of the exposure, even though previous studies have shown that for phthalates in particular, which are commonly found in personal and household care products, spot urine concentrations might still represent exposure up to 3 months prior, probably due to consistency in lifestyle (Casas et al., 2018). We identified associations of high exposure to certain, mainly more conventional, phthalates with a shorter time to pregnancy. This was an unexpected finding. This could be the effect of reversed causation. We aimed to enrol couples as soon as they started trying to conceive, but especially couples with prolonged attempts at pregnancy might have been enrolled later in the process. Potentially, those couples already adapted healthier lifestyle behaviours, thus having lower concentrations of the more well-known phthalates in urine. However, further studies are needed to replicate our findings and assess the underlying mechanisms. Some chemicals were only analyzed in one of our laboratories, therefore the sample size was smaller and thus the power to detect associations for this chemicals was lower. For our analysis on time to pregnancy and miscarriage, respectively 18 and 172 women participated twice in our study. Because of the non-persistency of our exposure, we included these women in our analysis. When we restricted the analyses to the first episode only of each women, we did observe similar results (data not shown). Last, due to the observational nature of the study, our results may be affected by residual confounding.

## 5. Conclusion

The current study does provide evidence of association of higher periconception exposure to certain bisphenols and phthalates with altered fecundity, in particular time to pregnancy, subfertility, and miscarriage, in the general population. It is among the first measuring bisphenol and phthalate concentrations in the preconception period in a large prospective cohort among the general population. Our results should be taken forward to future studies, potentially including emerging chemicals and long-term follow-up outcomes.

## Supplementary Material

1

Appendix A. Supplementary data

Supplementary data to this article can be found online at https://doi.org/10.1016/j.envres.2025.121712.

## Figures and Tables

**Table 1:** Characteristics of the study population.

	Population time to pregnancy and subfertility		Population miscarriage	
		
	Women (n = 760)	Men (n = 334)	Women (n = 1880)	Men (n = 1202)

Age in years, mean (±SD)	31–5 (4.0)	32.9 (4.7)	31–6 (4.2)	33.9 (5.3)
Ethnicity, n (%)				
Dutch	494 (64.7)	247 (75.8)	1165 (64.7)	798 (69.8)
European	75 (10.9)	28 (85–9)	190 (10–9)	106 (9.3)
Non-European	188 (24.3)	51 (15.6)	406 (24.3)	240 (21.0)
*Missing*	*3*	*8*	*119*	*58*
Highest education finished, n (%)				
Primary	5 (0.7)	2 (0.6)	12 (0.7)	16 (1.4)
Secondary	164 (21.8)	89 (26.8)	461 (26.4)	351 (30.6)
Higher	583 (77.5)	241 (72.6)	1276 (73.0)	779 (68.0)
*Missing*	*8*	*2*	*131*	*56*
Parity, n (%)		*NA*		*NA*
Nullipara	556 (75.2)		1096 (64.5)	
Multipara	183 (24.8)		603 (35.5)	
*Missing*	*21*		*181*	
Body mass index in kg/m^2^, median (95 % range)	23.7 (18.5, 38.9)	24.6 (19.8, 34.3)	23.2 (18.5, 35.6)	24.9 (19.8, 34.9)
*Missing*	*1*	*0*	*235*	*4*
Folic acid supplement use, n (%)	638 (99.5)	*NA*	1590 (99.0)	*NA*
*Missing*	*119*		*274*	
Smoking, n (%)		*NA*		*NA*
Never	418 (58–5)		860 (55.4)	
Quit before pregnancy	262 (36.7)		473 (30.5)	
Quit in first trimester of pregnancy	30 (4.2)		151 (9.7)	
Continued smoking inpregnancy	4 (0.6)		68 (4.4)	
*Missing*	*46*		*328*	
Alcohol use, n (%)		*NA*		*NA*
No	135 (18.4)		352 (20.9)	
< 3 months before pregnancy	499 (67.9)		1039 (61.6)	
In pregnancy	101 (13.7)		295 (17.5)	
*Missing*	*25*		*194*	
Laboratory of chemicals measurements, n (%)				
New York (NYU)	478 (62.9)	230 (68.9)	1181 (62.8)	790 (65.7)
Norway (NIPH)	282 (37.1)	104 (31.1)	699 (37.2)	412 (34.3)
Mode of conception, n (%)				
Spontaneous conception	469 (61.7)	281 (84.1)	1691 (89.9)	1097 (91.3)
Assisted conception, n (%)	108 (14.2)	53 (15.9)	189 (10.1)	105 (8.7)
No conception	183 (24.1)	0 (0.0)	0 (0.0)	0 (0.0)
Time to pregnancy, months, median (95 % range)	7.4 (0.0, 84.9)	6.0 (0.0, 66.4)	4.4 (0.0, 80.5)	3.9 (0.0, 65.0)
Duration pursuing pregnancy in women that did not conceive, months, median (95 % range)	29.6 (0.0, 43.7)	*NA*	*NA*	*NA*
Subfertility (time to pregnancy > 12 months or assisted reproduction), n %)	376 (51.6)	105 (31.4)	484 (30.0)	274 (25.0)
Miscarriage, n (%)	101 (13⋅3)	22 (6⋅6)	214 (11⋅4)	76 (6⋅3)
History of miscarriage, n (%)	147 (20⋅6)	*NA*	304 (19⋅9)	*NA*

Values presented as mean (SD), median (95 % range) or number of participants (valid %). NYU New York University NIPH Norwegian Institute of Public Health.

## Data Availability

Data will be made available on request.
