# Supplementary material for: Periconception bisphenol and phthalate concentrations in women and men, time to pregnancy, and risk of miscarriage
Source: Environ Res. Author manuscript; Available in PMC 2026 Aug 5. (PMC13440648; doi:10.1016/j.envres.2025.121712)
Supplement: Supplementary file 1 [file NIHMS2175012-supplement-1.docx]

**Supplemental Material**

**Periconception bisphenol and phthalate concentrations in women and men, time to pregnancy, and miscarriage.**

Sophia M Blaauwendraad, Aline J Boxem, Romy Gaillard, Linda G Kahn, Mathusa Lakuleswaran, Amrit Kaur Sakhi, Eline L Bekkers, Claudia J Kruithof, Zixuan Mo, Larry Spadacini, Cathrine Thomsen, Eric AP Steegers, Annemarie GMGJ Mulders, Vincent WV Jaddoe, Leonardo Trasande

**Text S1.** Harmonization of phthalates analyzed through isomer integration versus single peak integration methods.

**Text S2.** Details on grouping of the bisphenol and phthalate metabolites.

**Text S3.** Details on the Cox proportional hazards models on subfertility**.**

**Table S1.** Descriptive statistics of maternal chemicals including number of samples and percentage below the limit of detection (LOD) per chemical.

**Table S2.** Descriptive statistics of paternal chemicals including number of samples and percentage below the limit of detection (LOD) per chemical.

**Table S3.** Non-response analysis

**Table S4.** Characteristics of episodes with and without subfertility

**Table S5.** Characteristics of episodes with and without miscarriage

**Table S4.** Fecundability ratios for preconception individual and grouped urinary bisphenol and phthalate concentrations in women, basic models.

**Table S5.** Fecundability ratio for preconception individual and grouped urinary bisphenol and phthalate concentrations in women, adjusted models**.**

**Table S6.** Fecundability ratio for early pregnancy individual and grouped urinary bisphenol and phthalate concentrations in men, adjusted models**.**

**Table S7.** Associations of preconception individual and grouped urinary bisphenol and phthalate concentrations with subfertility in women, basic models.

**Table S8.** Associations of preconception individual and grouped urinary bisphenol and phthalate concentrations with subfertility in women, adjusted models.

**Table S9.** Associations of early pregnancy individual and grouped urinary bisphenol and phthalate concentrations with subfertility in men, adjusted models.

**Table S10.** Associations of periconception individual and grouped urinary bisphenol and phthalate concentrations with miscarriage in women, basic models.

**Table S11.** Associations of periconception individual and grouped urinary bisphenol and phthalate concentrations with miscarriage in women, adjusted models.

**Table S12.** Associations of early pregnancy individual and grouped urinary bisphenol and phthalate concentrations with miscarriage in men, adjusted models.

**Table S13.** Fecundability ratios for preconception individual and grouped urinary bisphenols and phthalate metabolites in women who conceived naturally (N = 652), adjusted models**.**

**Table S14.** Associations of preconception individual and grouped urinary bisphenol and phthalate concentrations with subfertility in women who conceived naturally(N=621), adjusted models.

**Table S15.** Associations of periconception individual and grouped urinary bisphenol and phthalate concentrations with miscarriage in women who conceived naturally (N=1691), adjusted models.

**Text S1.** Harmonization of phthalates analyzed through isomer integration versus single peak integration methods.

Four phthalate metabolites (Table 1) have multiple isomers due to long side chains, which can be poorly separated from each other. Thus, two different approaches can be used to integrate the isomers: (1) all the isomers are integrated together and (2) only one isomer for which internal standard is available is integrated. The Norwegian Institute of Public Health (NIPH) laboratory applied isomer integration, whereas the New York University (NYU) laboratory applied single peak integration.

| **Table 1. Phthalate metabolites with multiple isomers** | |
| --- | --- |
| **NIPH Lab Full Name (Abbreviation)** | **NYU Lab Full Name (Abbreviation)** |
| Mono-4-methyl-7-hydroxyoctyl phthalate (oh-MiNP) | Not analyzed |
| Mono-4-methyl-7-oxooctyl phthalate (oxo-MiNP) | Not analyzed |
| Mono-4-methyl-7-carboxyoctyl phthalate (cx-MiNP) | Mono-carboxy-isononyl phthalate (**mCINP**) |
| 6-Hydroxy Monopropylheptylphthalate (oh-MPHP) | Mono-2-(propyl-6-hydroxy-heptyl)-phthalate (**OH-MPHP**) |
| 2-(((Hydroxy-4-methyloctyl)oxy)carbonyl)cyclohexanecarboxylic Acid (oh-MINCH) | Cyclohexane-1,2-dicarboxylic acid-mono(hydroxy-isononyl) ester (**MHNCH**) |
| 2-(((4-Methyl-7-oxyooctyl)oxy)carbonyl)cyclohexanecarboxylic Acid (oxo-MINCH) | Cyclohexane-1,2-dicarboxylic acid-mono(oxo-isononyl) ester (**MONCH**) |

This difference in integration can result in different concentrations. Thus, to harmonize results between the two laboratories (NIPH and NYU), it was decided to calculate a ratio between multiple isomer integration and single isomer integration. To calculate these ratios, the NYU laboratory analyzed the urinary concentrations of those phthalates using both the single peak integration method and the isomer integration method in a random subset of urine samples (N = 600). The NYU laboratory used the same isomer integration approach as the NIPH laboratory. Subsequently, the single peak concentrations were regressed on the isomers concentration per phthalate, using the following equation:

$$\left[ isomer \right]=\beta0+ \beta1*\left[ single peak \right]+ \varepsilon$$

In which the β0 represents the intercept, the β1 the slope of the regression line and the ε the random error. We calculated the explained variance (r^2^) of the single peak concentration on the isomer concentration. We also obtained the standard error (SE) to express the degree of uncertainty in the accuracy of the dependent variables projected values. We subsequently used the β1 as the ratio to covert the single peak laboratory concentrations of the NYU phthalates to isomer concentrations, to make values comparable between the NYU and NIPH laboratory. We calculated the r^2^ and β1 per batch and overall, and concluded that those were consistent across batches. Therefore, we used the β1 calculated over all samples as ratio for conversion. The following isomers/batch were excluded: oh-MINCH/MHNCH from Batch 1, 2 and 3, and cx-MiNP/mCINP from Batch 3. For those isomers and batch, most samples were relatively integrated within isomer range. Therefore, inclusion of those values may misconstrue the data for other batches where it was originally integrated as a single peak.

**Table 2** summarizes the r^2^, β1, and SE per batch and overall.

The r^2^ was very high (≥0.9) for MCINP and MHNCH, and high (0.85 and 0.75) for MONCH and OH-MPHP. For MONCH and OH-MPHP, visual inspection of the regression plots showed that this r^2^ was mainly due to a few outliers, in which the isomer integration concentration was much higher as compared to the single peak integration concentration. Besides those, the overall agreement of the concentrations was high. The obtained ratios were 1.38 for MCINP, 1.32 for MHNCH, 1.37 for MONCH and 1.44 for OH-MPHP.

| **Table 2. Explained variance, ratio and standard error obtained from regression models regressing the single peak integration concentration of the chemicals on the isomer integration concentrations.** | | | | | | | | | | | | |
| --- | --- | --- | --- | --- | --- | --- | --- | --- | --- | --- | --- | --- |
|  | **MCINP** | | | **MHNCH** | | | **MONCH** | | | **OH-MPHP** | | |
|  | **Explained variance (R^2^)** | **Ratio** | **Standard Error (SE)** | **Explained variance (R^2^)** | **Ratio** | **Standard Error (SE)** | **Explained variance (R^2^)** | **Ratio** | **Standard Error (SE)** | **Explained variance (R^2^)** | **Ratio** | **Standard Error (SE)** |
| **Batch 1&2** | 0·95 | 1·09 | 0·08 | - | - | - | 0·80 | 1·14 | 0·42 | 0·92 | 1·05 | 0·18 |
| **Batch 3** | - | - | - | - | - | - | 0·80 | 1·03 | 0·44 | 0·81 | 2·11 | 0·15 |
| **Batch 4** | 0·96 | 1·60 | 0·12 | 1·00 | 1·04 | 0·13 | 0·93 | 1·33 | 0·35 | 0·78 | 1·93 | 0·44 |
| **Batch 5** | 0·96 | 1·35 | 0·09 | 0·97 | 1·79 | 0·75 | 0·96 | 1·86 | 0·30 | 0·83 | 2·01 | 0·24 |
| **Batch 6** | 0·93 | 1·74 | 0·13 | 0·96 | 1·77 | 0·51 | 0·92 | 2·13 | 0·24 | 0·79 | 1·43 | 0·64 |
| **All samples** | 0·91 | 1·38 | 0·15 | 0·90 | 1·32 | 1·14 | 0·85 | 1·37 | 0·45 | 0·75 | 1·44 | 0·43 |
| Values are obtained from the following regression models: [isomer] = β0 + β1 * [single peak] + ε, in which the slope (β1) is considered the ratio between the single peak and isomer integration concentrations of the chemicals. Values below the limit of detection have been replaced by the limit of detection (MCINP 0.15 ng/mL, MHNCH 0.02 ng/mL, MONCH 0.02 ng/mL, OH-MPHP 0.01 ng/mL).  MCINP mono-carboxy-isononyl phthalate, MHNCH cyclohexane-1,2-dicarboxylic acid-mono(hydroxy-isononyl) ester, MONCH cyclohexane-1,2-dicarboxylic acid-mono(oxo-isononyl) ester, OH-MPHP mono-2-(propyl-6-hydroxy-heptyl)-phthalate. | | | | | | | | | | | | |

Thus, we have used those overall ratio’s in and multiplied them with the concentrations of the samples measured at NYU.

**Text S2.** Details on grouping of the bisphenol and phthalate metabolites.

We converted the urinary concentrations of the individual bisphenols and phthalates from ng/mL to nmol/L using the molecular weight, and subsequently calculated the weighted molar sums for each bisphenol and phthalate group. For the total bisphenol (BP), low-molecular-weight (LMW), high-molecular-weight (HMW), and di-2-ethylhexyl (DEHP) group, we only included chemicals that were measured in both laboratories. As all di-n-ocyl phthalate (DNOP) chemicals were only measured at the NYU, we included all chemicals that were measured there. Thus, the BP group consisted of bisphenol A (BPA) and bisphenol S (BPS). The LMW group consisted of mono-ethyl phthalate (mEP), mono-iso-butyl phthalate (mIBP), and mono-n-butyl phthalate (mnBP). The DEHP group consisted of mono-2-ethylhexyl phthalate (mEHP), mono-2-ethyl 5-carboxypentyl phthalate (mECPP), mono-2-ethyl-5-hydroxyhexyl phthalate (mEHHP ), and mono-2-ethyl-5-oxohexyl phthalate (mEOHP). The DNOP group consisted of mono(3-carboxypropyl)phthalate (mCPP) and mono-(7-carboxy-n-heptyl)phthalate (mCHpP).

| **Table 1. Grouping of the bisphenols and phthalates** | |
| --- | --- |
| **Group** | **Individual metabolites** |
| Total bisphenol (BP) | Bisphenol A (BPA)  Bisphenol S (BPS) |
| Low-molecular-weight phthalate (LMW) | Mono-ethyl phthalate (mEP)  Mono-iso-butyl phthalate (mIBP)  Mono-n-butyl phthalate (mnBP) |
| Di-2-ethylhexyl phthalate | Mono-2-ethylhexyl phthalate (mEHP)  Mono-2-ethyl 5-carboxypentyl phthalate (mECPP)  Mono-2-ethyl-5-hydroxyhexyl phthalate (mEHHP )  Mono-2-ethyl-5-oxohexyl phthalate (mEOHP) |
| Di-n-ocyl phthalate (DNOP) | Mono(3-carboxypropyl)phthalate (mCPP)  Mono-(7-carboxy-n-heptyl)phthalate (mCHpP) |

**Text S3.** Details on the Cox proportional hazards models on subfertility

We assessed the associations of individual and grouped maternal preconception chemicals and fecundability using Cox proportional hazard models (R package *survival*). The survival outcome of this model was conception or no conception. The time variable was based on time to pregnancy, or the duration of actively pursuing pregnancy among women without conception. We checked the proportional hazard assumptions of the covariates using the Schoenfeld residuals, assessed linearity of all associations using the Martingale residuals, and assessed influential variables using the deviance residuals. For each chemical exposure, the HR was calculated by dividing the hazard rate of conceiving within one month with one IQR increase of the chemical by the hazard rate of the mean chemical concentration, $HR= \frac{H\left( t \right) +1 IQR [chemical]}{H\left( t \right) [chemical]}$. The resulting HR from the Cox model represents the fecundability ratio, representing the odds on conceiving per menstrual cycle. A HR <1 indicates a prolonged time per pregnancy per IQR increase in chemical exposure.

| **Table S1.** Descriptive statistics of women chemicals including number of samples and percentage below the limit of detection (LOD) per chemical. | | | | | | | | |
| --- | --- | --- | --- | --- | --- | --- | --- | --- |
| **Chemicals** | **Samples, n** | **Median (95% CI), ng/mL** | **Median (95% CI), ng/mL, NYU** | **Median (95% CI), ng/mL, NIPH** | **LOD NYU** | **LOD NIPH** | **Below LOD, n** | **Below LOD, %** |
| Phthalic acid (PA) | 1443 | 40·9 (0·78, 456·36) | 40.9 (0.78, 456.36) | - | 1.11 | - | 213 | 14.8* |
| **Low molecular weight phthalates** | | | | | | | | |
| Mono-methyl phthalate (mMP) | 1443 | 0·04 (0·04, 10·38) | 0.04 (0.04, 10.38) | - | 0.06 | - | 1076 | 74.6* |
| Mono-ethyl phthalate (mEP) | 2304 | 17·1 (1·04, 729·85) | 21.7 (1.13, 788.8) | 11.55 (0.92, 499.57) | 0.06 | 0.20 | 2 | 0.1* |
| Mono-iso-butyl phthalate (mIBP) | 2304 | 8·57 (0·64, 87·03) | 9.65 (0.77, 92.39) | 7.16 (0.48, 67.97) | 0.09 | 0.20 | 13 | 0.6* |
| Mono-n-butyl phthalate (mnBP) | 1443 | 6·67 (0·5, 55·47) | 7.36 (0.52, 59.59) | 5.82 (0.45, 45.98) | 0.14 | 0.20 | 28 | 1.2* |
| **High molecular weight phthalates** | | | | | | | | |
| **Di-2-ethylhexyl phthalate** | | | | | | | | |
| Mono-2-ethylhexyl phthalate (mEHP) | 2304 | 0·97 (0·14, 10·27) | 1.57 (0.21, 11.98) | 0.34 (0.14, 5.62) | 0.30 | 0.20 | 692 | 30.0* |
| Mono-2-ethyl 5-carboxypentyl phthalate (mECPP) | 2304 | 4·38 (0·21, 24·79) | 3.96 (0.21, 26.37) | 4.96 (1.98, 23.25) | 0.29 | 0.70 | 67 | 2.9* |
| Mono-[(2-carboxymethyl)hexyl]phthalate (mCMHP) | 1443 | 1·09 (0·03, 9·55) | 1.09 (0.03, 9.55) | - | 0.04 | - | 1145 | 79.3* |
| Mono-2-ethyl-5-hydroxyhexyl phthalate (mEHHP ) | 2304 | 3·54 (0·41, 25·64) | 4.3 (0.39, 30.29) | 2.67 (0.43, 16.29) | 0.08 | 0.20 | 5 | 0.2* |
| Mono-2-ethyl-5-oxohexyl phthalate (mEOHP) | 2304 | 1·89 (0·21, 12·28) | 2.08 (0.2, 14.08) | 1.64 (0.23, 10.23) | 0.04 | 0.20 | 22 | 1.0* |
| ***Di-n-octyl phthalate (DNOP)*** | | | | | | | | |
| Mono-n-octyl phthalate (mOP) | 2304 | 0·18 (0·18, 0·35) | 0.18 (0.18, 0.18) | 0.35 (0.35, 0.35) | 0.25 | 0.50 | 2285 | 99.2 |
| Mono(3-carboxypropyl)phthalate (mCPP) | 1443 | 0·01 (0·01, 2·78) | 0.01 (0.01, 2.78) | - | 0.01 | - | 765 | 53.0* |
| Mono-(7-carboxy-n-heptyl)phthalate (mCHpP) | 1443 | 0·04 (0·04, 1·65) | 0.04 (0.04, 1.65) | - | 0.06 | - | 973 | 67.4* |
| ***Diisononyl phthalate (DINP)*** | | | | | | | | |
| Monoisononylphthalate (mINP) | 1443 | 0·13 (0·13, 0·13) | 0.13 (0.13, 0.13) | - | 0.18 | - | 1443 | 100.0 |
| Mono-carboxy-isoctyl phthalate (mCIOP) | 1443 | 1·24 (0·14, 10·2) | 1.24 (0.14, 10.2) | - | 0.20 | - | 231 | 16.0* |
| Mono-4-methyl-7-hydroxyoctyl phthalate (ohMiNP) | 861 | 2·31 (0·21, 23·91) | 2.12 (0.58, 11.55) | 2.31 (0.21, 24.32) | - | 0.20 | 4 | 0.5* |
| Mono-4-methyl-7-oxooctyl phthalate (oxoMiNP) | 861 | 1·17 (0·14, 13·03) | 1.33 (0.26, 8.66) | 1.16 (0.14, 13.06) | - | 0.20 | 129 | 15.0* |
| Mono-4-methyl-7-carboxyoctyl phthalate (cxMiNP/mCINP) | 2304 | 1·92 (0·23, 15·77) | 0.46 (0.22, 4) | 3.73 (1.58, 28.02) | 0.15 | 0.70 | 700 | 30.4* |
| ***Di(isononyl)cyclohexane-1,2-dicarboxylate (DINCH)*** | | | | | | | | |
| Cyclohexane-1,2-dicarboxylic acid-monocarboxy isooctyl ester (mCOCH) | 1443 | 0·2 (0·01, 2·63) | 0.2 (0.01, 2.63) | - | 0.02 | - | 181 | 12.5* |
| 2-(((Hydroxy-4-methyloctyl)oxy)carbonyl)cyclohexanecarboxylic Acid (ohMINCH/mHNCH) | 2304 | 0·72 (0·01, 14·84) | 0.73 (0.01, 16.33) | 0.68 (0.14, 11.75) | 0.02 | 0.20 | 257 | 11.2* |
| 2-(((4-Methyl-7-oxyooctyl)oxy)carbonyl)cyclohexanecarboxylic Acid (oxoMINCH/mONCH) | 2304 | 0·23 (0, 4·6) | 0.35 (0.05, 5.25) | 0.23 (0.14, 5.64) | 0.02 | 0.20 | 407 | 17.7* |
| ***Di-propyl-heptyl phthalate (DiDP/DPHP)*** | | | | | | | | |
| Mono-(8-methyl-1-nonyl)phthalate (mIDP) | 1443 | 0·63 (0·63, 0·63) | 0.63 (0.63, 0.63) | - | 0.89 | - | 1420 | 98.4 |
| Mono-2-(propyl-6-oxoheptyl)-phthalate (oxomPHP) | 1443 | 0·16 (0·01, 1·77) | 0.16 (0.01, 1.77) | - | 0.01 | - | 300 | 20.8* |
| Mono-2-(propyl-6-carboxy-hexyl)-phthalate (cxmPHxP) | 1443 | 0·03 (0·01, 0·61) | 0.03 (0.01, 0.61) | - | 0.02 | - | 676 | 46.8* |
| 6-Hydroxy monopropylheptylphthalate (OHmPHP) | 2304 | 0·14 (0·01, 1·96) | 0.11 (0.01, 1.69) | 0.23 (0.14, 2.12) | 0.01 | 0.20 | 937 | 40.7* |
| **Other** | | | | | | | | |
| Mono benzyl phthalate (mBzBP) | 2304 | 0·22 (0·05, 16·71) | 0.11 (0.11, 20.07) | 0.51 (0.05, 9.25) | 0.15 | 0.07 | 1061 | 46.1* |
| Mono-hexylphthalate (mHxP) | 1443 | 0·04 (0·04, 0·67) | 0.04 (0.04, 0.67) | - | 0.06 | - | 1087 | 75.3* |
| Mono-2-heptylphthalate (mHpP) | 1443 | 0·21 (0·21, 0·21) | 0.21 (0.21, 0.21) | - | 0.30 | - | 1426 | 98.8 |
| Mono-n-pentyl phthalate (MnPeP) | 861 | 0·14 (0·14, 0·14) | - | 0.14 (0.14, 0.14) | - | 0.20 | 349 | 40.5* |
| Monocyclohexyl phthalate (mCHP) | 2304 | 0·03 (0·03, 0·14) | 0.03 (0.03, 0.03) | 0.14 (0.14, 0.14) | 0.04 | 0.20 | 2273 | 98.7 |
| **Bisphenols** | | | | | | | | |
| BPA | 2304 | 0·98 (0·11, 18·28) | 0.6 (0.11, 7.48) | 2.14 (0.03, 27.74) | 0.152 | 0.04 | 449 | 19.5* |
| BPS | 2304 | 0·22 (0·04, 4·79) | 0.39 (0.04, 5.73) | 0.07 (0.07, 3.12) | 0.05 | 0.10 | 806 | 35.0* |
| BPZ | 1443 | 0·29 (0·29, 0·29) | 0.29 (0.29, 0.29) | - | 0.41 | - | 1443 | 100.0 |
| BPB | 2304 | 0·07 (0·02, 0·07) | 0.07 (0.07, 0.07) | 0.02 (0.02, 0.02) | 0.10 | 0.03 | 2293 | 99.5 |
| BPF | 2304 | 0·14 (0·13, 5·34) | 0.13 (0.13, 5.93) | 0.14 (0.14, 4.56) | 0.18 | 0.20 | 1891 | 82.1 |
| BPAP | 1443 | 0·05 (0·05, 1·2) | 0.05 (0.05, 1.2) | - | 0.07 | - | 1286 | 89.1 |
| BPAF | 2304 | 0·56 (0·03, 0·56) | 0.56 (0.56, 0.56) | 0.03 (0.03, 0.03) | 0.79 | 0.04 | 2303 | 100.0 |
| BPP | 1443 | 0·08 (0·08, 0·08) | 0.08 (0.08, 0.08) | - | 0.11 | - | 1437 | 99.6 |
| LOD limit of detection, NYU New York University, NIPH Norwegian Institute of Public Health *<80% below LOD and therefore included in analyses. Values <LOD have been imputed by LOD divided by the square root of 2. | | | | | | | | |

| **Table S2.** Descriptive statistics of men chemicals including number of samples and percentage below the limit of detection (LOD) per chemical. | | | | | | |
| --- | --- | --- | --- | --- | --- | --- |
| **Chemicals** | **Samples, n** | **Median (95% CI), ng/mL** | **LOD NYU** | **LOD NIPH** | **Below LOD, n** | **Below LOD, %** |
| Phthalic acid (PA) | 827 | 28·4 (0·78, 371·35) | 1.11 | - | 149 | 18.0* |
| **Low molecular weight phthalates** | | | | | | |
| Mono-methyl phthalate (mMP) | 827 | 0·04 (0·04, 13·83) | 0.06 | - | 658 | 79.6* |
| Mono-ethyl phthalate (mEP) | 1256 | 13·8 (1·19, 553·96) | 0.06 | 0.20 | 2 | 0.2* |
| Mono-iso-butyl phthalate (mIBP) | 1256 | 8·29 (0·7, 68·82) | 0.09 | 0.20 | 8 | 0.6* |
| Mono-n-butyl phthalate (mnBP) | 1256 | 6·5 (0·1, 44·66) | 0.14 | 0.20 | 44 | 3.5* |
| **High molecular weight phthalates** | | | | | | |
| **Di-2-ethylhexyl phthalate** | | | | | | |
| Mono-2-ethylhexyl phthalate (mEHP) | 1256 | 1·24 (0·14, 9·44) | 0.30 | 0.20 | 320 | 25.5* |
| Mono-2-ethyl 5-carboxypentyl phthalate (mECPP) | 1256 | 3·82 (0·32, 26·76) | 0.29 | 0.70 | 31 | 2.5* |
| Mono-[(2-carboxymethyl)hexyl]phthalate (mCMHP) | 827 | 0·93 (0·03, 13·91) | 0.04 | - | 574 | 69.4* |
| Mono-2-ethyl-5-hydroxyhexyl phthalate (mEHHP ) | 1256 | 3·86 (0·5, 25·05) | 0.08 | 0.20 | 2 | 0.2* |
| Mono-2-ethyl-5-oxohexyl phthalate (mEOHP) | 1256 | 1·78 (0·23, 10·27) | 0.04 | 0.20 | 8 | 0.6* |
| ***Di-n-octyl phthalate (DNOP)*** | | | | | | |
| Mono-n-octyl phthalate (mOP) | 1256 | 0·18 (0·18, 0·35) | 0.25 | 0.50 | 1247 | 99.3 |
| Mono(3-carboxypropyl)phthalate (mCPP) | 827 | 0·1 (0·01, 3·39) | 0.01 | - | 361 | 43.7* |
| Mono-(7-carboxy-n-heptyl)phthalate (mCHpP) | 827 | 0·04 (0·04, 0·66) | 0.06 | - | 747 | 90.3 |
| ***Diisononyl phthalate (DINP)*** | | | | | | |
| Monoisononylphthalate (mINP) | 827 | 0·13 (0·13, 0·13) | 0.18 | - | 827 | 100.0 |
| Mono-carboxy-isoctyl phthalate (mCIOP) | 827 | 1·81 (0·27, 19·56) | 0.20 | - | 13 | 1.6* |
| Mono-4-methyl-7-hydroxyoctyl phthalate (ohMiNP) | 429 | 2·5 (0·31, 17·24) | - | 0.20 | 3 | 0.7* |
| Mono-4-methyl-7-oxooctyl phthalate (oxoMiNP) | 429 | 1·18 (0·14, 11·92) | - | 0.20 | 42 | 9.8* |
| Mono-4-methyl-7-carboxyoctyl phthalate (cxMiNP/mCINP) | 1256 | 1·08 (0·23, 11·79) | 0.15 | 0.70 | 289 | 23.0* |
| ***Di(isononyl)cyclohexane-1,2-dicarboxylate (DINCH)*** | | | | | | |
| Cyclohexane-1,2-dicarboxylic acid-monocarboxy isooctyl ester (mCOCH) | 827 | 0·28 (0·03, 3·71) | 0.02 | - | 13 | 1.6* |
| 2-(((Hydroxy-4-methyloctyl)oxy)carbonyl)cyclohexanecarboxylic Acid (ohMINCH/mHNCH) | 1256 | 0·99 (0·03, 16·05) | 0.02 | 0.20 | 88 | 7.0* |
| 2-(((4-Methyl-7-oxyooctyl)oxy)carbonyl)cyclohexanecarboxylic Acid (oxoMINCH/mONCH) | 1256 | 0·26 (0, 5) | 0.02 | 0.20 | 164 | 13.1* |
| ***Di-propyl-heptyl phthalate (DiDP/DPHP)*** | | | | | | |
| Mono-(8-methyl-1-nonyl)phthalate (mIDP) | 827 | 0·63 (0·63, 0·63) | 0.89 | - | 827 | 100.0 |
| Mono-2-(propyl-6-oxoheptyl)-phthalate (oxomPHP) | 827 | 0·19 (0·01, 1·69) | 0.01 | - | 98 | 11.9* |
| Mono-2-(propyl-6-carboxy-hexyl)-phthalate (cxmPHxP) | 827 | 0·09 (0·01, 0·63) | 0.02 | - | 244 | 29.5* |
| 6-Hydroxy monopropylheptylphthalate (OHmPHP) | 1256 | 0·37 (0·04, 3·89) | 0.01 | 0.20 | 292 | 23.2* |
| **Other** | | | | | | |
| Mono benzyl phthalate (mBzBP) | 1256 | 0·25 (0·05, 15·43) | 0.15 | 0.07 | 561 | 44.7* |
| Mono-hexylphthalate (mHxP) | 827 | 0·04 (0·04, 0·88) | 0.06 | - | 539 | 65.2* |
| Mono-2-heptylphthalate (mHpP) | 827 | 0·21 (0·21, 0·21) | 0.30 | - | 814 | 98.4 |
| Mono-n-pentyl phthalate (MnPeP) | 429 | 0·14 (0·14, 0·14) | - | 0.20 | 426 | 99.3 |
| Monocyclohexyl phthalate (mCHP) | 1256 | 0·03 (0·03, 0·18) | 0.04 | 0.20 | 1213 | 96.6 |
| **Bisphenols** | | | | | | |
| BPA | 1256 | 0·66 (0·03, 10·32) | 0.152 | 0.04 | 400 | 31.8* |
| BPS | 1256 | 0·23 (0·04, 5·67) | 0.05 | 0.10 | 457 | 36.4* |
| BPZ | 827 | 0·14 (0·13, 8·27) | 0.41 | - | 824 | 99.6 |
| BPB | 1256 | 0·29 (0·29, 0·29) | 0.10 | 0.03 | 1255 | 99.9 |
| BPF | 1256 | 0·08 (0·08, 0·08) | 0.18 | 0.20 | 829 | 66.0* |
| BPAP | 827 | 0·56 (0·03, 0·56) | 0.07 | - | 685 | 82.8 |
| BPAF | 1256 | 0·05 (0·05, 1·15) | 0.79 | 0.04 | 1256 | 100.0 |
| BPP | 827 | 0·07 (0·02, 0·07) | 0.11 | - | 827 | 100.0 |
| LOD limit of detection, NYU New York University, NIPH Norwegian Institute of Public Health *<80% below LOD and therefore included in analyses. Values <LOD have been imputed by LOD divided by the square root of 2. | | | | | | |

| **Table S3.** Non-response analysis of characteristics of episodes included and excluded from the study population | | | | | | |
| --- | --- | --- | --- | --- | --- | --- |
|  | **Women** | | | **Men** | | |
|  | **Non-responders**  **(n = 211)** | **Responders**  **(n = 2093)** | **p-value** | **Non-responders**  **(n = 43)** | **Responders**  **(n = 1213)** | **p-value** |
| Age in years, mean (±SD) | 31·0 (4·9) | 31·8 (4·3) | *0·019* | 31·9 (4·6) | 33·9 (5·3) | *0·013* |
| *Missing* | *0* | *0* |  | *0* | *0* |  |
| Ethnicity, n (%) |  |  |  |  |  |  |
| Dutch | 90 (61·6) | 1282 (65·0) | *0·705* | 25 (75·8) | 805 (69·7) | *0·708* |
| European | 18 (12·3) | 214 (10·8) |  | 3 (9·1) | 108 (9·4) |  |
| Non-European | 38 (26·0) | 477 (24·2) |  | 5 (15·2) | 242 (21·0) |  |
| *Missing* | *65* | *120* |  | *10* | *58* |  |
| Highest education finished, n (%) |  |  |  |  |  |  |
| Primary | 2 (1·4) | 14 (0·7) |  | 0 (0·0) | 16 (1·4) | *0·467* |
| Secondary | 41 (28·1) | 523 (26·7) |  | 13 (39·4) | 354 (30·6) |  |
| Higher | 103 (70·5) | 1424 (72·6) |  | 20 (60·6) | 787 (68·0) |  |
| *Missing* | *65* | *132* |  | *10* | *56* |  |
| Parity, n (%) |  |  |  |  |  |  |
| Nullipara | 104 (73·8) | 1255 (66·1) | *0·075* | NA | NA |  |
| Multipara | 37 (26·2) | 645 (33·9) |  |  |  |  |
| *Missing* | *70* | *193* |  |  |  |  |
| Body mass index in kg/m^2^, median (95% range) | 23·7 [18·2, 37·5] | 23·3 [18·5, 36·6] | *0·108* | 25·4 [18·8, 30·4] | 24·9 [19·8, 34·9] | *0·467* |
| *Missing* | *34* | *235* |  | *1* | *4* |  |
| Folic acid supplement use, n (%) |  |  |  |  |  |  |
| No | 0 (0·0) | 16 (0·9) | *0·980* | NA | NA |  |
| Yes | 58 (100·0) | 1718 (99·1) |  |  |  |  |
| *Missing* | *153* | *359* |  |  |  |  |
| Smoking, n (%) |  |  |  |  |  |  |
| Never | 58 (53·2) | 971 (55·4) | *0·146* | NA | NA |  |
| Quit before pregnancy | 38 (34·9) | 562 (32·1) |  |  |  |  |
| Quit in first trimester of pregnancy | 5 (4·6) | 152 (8·7) |  |  |  |  |
| Continued smoking in pregnancy | 8 (7·3) | 68 (3·9) |  |  |  |  |
| *Missing* | *102* | *340* |  |  |  |  |
| Alcohol use, n (%) |  |  |  |  |  |  |
| No | 22 (20·6) | 386 (20·5) | *0·006* | NA | NA |  |
| <3 months before pregnancy | 80 (74·8) | 1199 (63·6) |  |  |  |  |
| In pregnancy | 5 (4·7) | 299 (15·9) |  |  |  |  |
| *Missing* | *104* | *209* |  |  |  |  |
| Laboratory of chemicals measurements, n (%) |  |  |  |  |  |  |
| New York (NYU) | 134 (63·5) | 1309 (62·5) | *0·840* | 32 (74·4) | 795 (65·5) | *0·297* |
| Norway (NIPH) | 77 (36·5) | 784 (37·5) |  | 11 (25·6) | 418 (34·5) |  |
| *Missing* | *0* | *0* |  | *0* | *0* |  |
| Mode of conception, n (%) |  |  |  |  |  |  |
| Spontaneous conception | 79 (37·4) | 1709 (81·7) | *<0·001* | 40 (93·0) | 1106 (91·2) | *1·00* |
| Assisted conception | 6 (2·8) | 201 (9·6) |  | 3 (7·0) | 107 (8·8) |  |
| No conception | 125 (59·2) | 183 (8·7) |  | 0 | 0 |  |
| Time to pregnancy, months, median (95% range) | 3·4 [0·0, 55·6] | 4·5 [0·0, 81·0] |  | 2·8 [0·0, 59·8] | 4·0 [0·0, 64·7] |  |
| *Missing* | *158* | *460* |  | *14* | *109* |  |
| Duration pursuing pregnancy in women that did not conceive, months, median (95% range) | NA | 128·1  [20·9, 519·9] |  | NA | NA |  |
| *Missing* | *211* | *1910* |  | *43* | *1213* |  |
| Subfertility (time to pregnancy >12 months or assisted reproduction), n (%) |  |  |  |  |  |  |
| No | 40 (74·1) | 1141 (63·5) | *0·148* | 24 (82·8) | 828 (74·9) | *0·452* |
| Yes | 14 (25·9) | 655 (36·5) |  | 5 (17·2) | 278 (25·1) |  |
| *Missing* | *157* | *297* |  | *14* | *107* |  |
| Miscarriage, n (%) |  |  |  |  |  |  |
| No | 211 (100·0) | 1879 (89·8) | *<0·001* | 43 (100·0) | 1137 (93·7) | *0·171* |
| Yes | 0 (0·0) | 214 (10·2) |  | 0 (0·0) | 76 (6·3) |  |
| *Missing* | *0* | *0* |  | *0* | *0* |  |
| History of miscarriage, n (%) |  |  |  |  |  |  |
| No | 92 (85·2) | 1388 (80·2) | *0·256* | NA | NA |  |
| Yes | 16 (14·8) | 342 (19·8) |  |  |  |  |
| *Missing* | *103* | *363* |  |  |  |  |
| Values presented as mean (SD), median (95% range) or number of participants (valid %)· NYU New York University NIPH Norwegian Institute of Public Health· | | | | | | |

| **Table S4.** Characteristics of episodes with and without subfertility | | | | | | |
| --- | --- | --- | --- | --- | --- | --- |
|  | **Women** | | | **Men** | | |
|  | **Fertile**  **(n = 353)** | **Subfertile**  **(n = 376)** | **p-value** | **Fertile**  **(n = 229)** | **Subfertile**  **(n = 105)** | **p-value** |
| Age in years, mean (±SD) | 31.5 (3.4) | 32.5 (4.6) | 0.002 | 33.2 (4.6) | 34.8 (4.9) | 0.003 |
| *Missing* |  |  |  |  |  |  |
| Ethnicity, n (%) |  |  |  |  |  |  |
| Dutch | 255 (72.2) | 215 (57.6) | <0.001 | 169 (75.8) | 78 (75.7) | 0.351 |
| European | 35 (9.9) | 35 (9.4) |  | 22 (9.9) | 6 (5.8) |  |
| Non-European | 63 (17.8) | 123 (33.0) |  | 32 (14.3) | 19 (18.4) |  |
| *Missing* | *0* | *3* |  | *6* | *2* |  |
| Highest education finished, n (%) |  |  |  |  |  |  |
| Primary | 1 (0.3) | 4 (1.1) | <0.001 | 2 (0.9) | 0 (0.0) | 0.036 |
| Secondary | 52 (15.0) | 107 (28.5) |  | 52 (22.8) | 37 (35.6) |  |
| Higher | 293 (84.7) | 264 (70.4) |  | 174 (76.3) | 67 (64.4) |  |
| *Missing* | *7* | *1* |  | *1* | *1* |  |
| Parity, n (%) |  |  |  |  |  |  |
| Nullipara | 263 (75.1) | 266 (74.1) | 263 (75.1) | NA | NA |  |
| Multipara | 87 (24.9) | 93 (25.9) | 87 (24.9) |  |  |  |
| *Missing* | *3* | *17* |  |  |  |  |
| Body mass index in kg/m^2^, median (95% range) | 23.3 [18.5-38.0] | 24.4 [18.5-40.8] |  | 24.4 [19.6-31.8] | 25.2 [21.1-36.7] |  |
| *Missing* | *1* | *0* |  | *0* | *0* |  |
| Folic acid supplement use, n (%) |  |  |  |  |  |  |
| No | 1 (0.3) | 2 (0.7) | 0.893 | NA | NA |  |
| Yes | 333 (99.7) | 284 (99.3) |  |  |  |  |
| *Missing* | *19* | *90* |  |  |  |  |
| Smoking, n (%) |  |  |  |  |  |  |
| Never | 213 (63.2) | 186 (53.6) | <0.001 | NA | NA |  |
| Quit before pregnancy | 101 (30.0) | 150 (43.2) |  |  |  |  |
| Quit in first trimester of pregnancy | 22 (6.5) | 8 (2.3) |  |  |  |  |
| Continued smoking in pregnancy | 1 (0.3) | 3 (0.9) |  |  |  |  |
| *Missing* | *16* | *29* |  |  |  |  |
| Alcohol use, n (%) |  |  |  |  |  |  |
| No | 59 (17.0) | 75 (20.9) | <0.001 | NA | NA |  |
| <3 months before pregnancy | 212 (60.9) | 259 (72.3) |  |  |  |  |
| In pregnancy | 77 (22.1) | 24 (6.7) |  |  |  |  |
| *Missing* | *5* | *18* |  |  |  |  |
| Laboratory of chemicals measurements, n (%) |  |  |  |  |  |  |
| New York (NYU) | 219 (62.0) | 235 (62.5) | 0.959 | 159 (69.4) | 71 (67.6) | 0.838 |
| Norway (NIPH) | 134 (38.0) | 141 (37.5) |  | 70 (30.6) | 34 (32.4) |  |
| *Missing* | *0* | *0* |  | *0* | *0* |  |
| Mode of conception, n (%) |  |  |  |  |  |  |
| Spontaneous conception | 353 (100.0) | 116 (30.9) |  | 229 (100.0) | 52 (49.5) |  |
| Assisted conception | 0 | 108 (28.7) |  | 0 | 53 (50.5) |  |
| No conception | 0 | 152 (40.4) |  | 0 | 0 |  |
| Time to pregnancy, months, median (95% range) | 3.7 [0.0-11.4] | 25.3 [12.6-112.0] |  | 3.7 [0.0-11.4] | 23.2 [12.4-86.7] |  |
| *Missing* | *0* | *152* |  | *0* | *0* |  |
| Duration pursuing pregnancy in women that did not conceive, months, median (95% range) | NA | 141.4  [53.9-532.0] |  | NA | NA |  |
| *Missing* | *353* | *224* |  | *229* | *105* |  |
| Subfertility (time to pregnancy >12 months or assisted reproduction), n (%) |  |  |  |  |  |  |
| No | 353 (100.0) | 0 (0.0) | <0.001 | 229 (100.0) | 0 (0.0) | <0.001 |
| Yes | 0 (0.0) | 376 (100.0) |  | 0 (0.0) | 105 (100.0) |  |
| *Missing* | *0* | *0* |  | *0* | *0* |  |
| Miscarriage, n (%) |  |  |  |  |  |  |
| No | 298 (84.4) | 330 (87.8) | 0.230 | 214 (93.4) | 98 (93.3) | 1.000 |
| Yes | 55 (15.6) | 46 (12.2) |  | 15 (6.6) | 7 (6.7) |  |
| *Missing* | *0* | *0* |  | *0* | *0* |  |
| History of miscarriage, n (%) |  |  |  |  |  |  |
| No | 284 (83.5) | 255 (74.3) | 0.004 | NA | NA |  |
| Yes | 56 (16.5) | 88 (25.7) |  |  |  |  |
| *Missing* | *13* | *33* |  |  |  |  |
| Values presented as mean (SD), median (95% range) or number of participants (valid %)· NYU New York University NIPH Norwegian Institute of Public Health· | | | | | | |

| **Table S5.** Characteristics of episodes with and without miscarriage | | | | | | |
| --- | --- | --- | --- | --- | --- | --- |
|  | **Women** | | | **Men** | | |
|  | **No miscarriage**  **(n = 1666)** | **Miscarriage**  **(n = 214)** | **p-value** | **No miscarriage**  **(n = 1126)** | **Miscarriage**  **(n = 76)** | **p-value** |
| Age in years, mean (±SD) | 31.6 (4.1) | 32.9 (4.4) | <0.001 | 33.8 (5.2) | 35.8 (6.3) | 0.002 |
| *Missing* | *0* | *0* |  | *0* | *0* |  |
| Ethnicity, n (%) |  |  |  |  |  |  |
| Dutch | 1038 (66.0) | 127 (67.2) | 0.380 | 748 (69.6) | 50 (72.5) | 0.877 |
| European | 175 (11.1) | 15 (7.9) |  | 100 (9.3) | 6 (8.7) |  |
| Non-European | 359 (22.8) | 47 (24.9) |  | 227 (21.1) | 13 (18.8) |  |
| *Missing* | *94* | *25* |  | *51* | *7* |  |
| Highest education finished, n (%) |  |  |  |  |  |  |
| Primary | 11 (0.7) | 1 (0.5) | 0.913 | 15 (1.4) | 1 (1.4) | 0.018 |
| Secondary | 409 (26.2) | 52 (27.4) |  | 319 (29.6) | 32 (45.7) |  |
| Higher | 1139 (73.1) | 137 (72.1) |  | 742 (69.0) | 37 (52.9) |  |
| *Missing* | *107* | *24* |  | *50* | *6* |  |
| Parity, n (%) |  |  |  |  |  |  |
| Nullipara | 973 (64.3) | 123 (66.5) | 0.607 | NA | NA |  |
| Multipara | 541 (35.7) | 62 (33.5) |  |  |  |  |
| *Missing* | *152* | *29* |  |  |  |  |
| Body mass index in kg/m^2^, median (95% range) | 23.1 [18.6-35.7] | 23.9 [18.1-34.9] |  | 24.9 [19.8-34.9] | 25.1 [19.5-35.0] |  |
| *Missing* | *193* | *42* |  | *4* | *0* |  |
| Folic acid supplement use, n (%) |  |  |  |  |  |  |
| No | 15 (1.0) | 1 (0.7) | 1.000 | NA | NA |  |
| Yes | 1443 (99.0) | 147 (99.3) |  |  |  |  |
| *Missing* | *208* | *66* |  |  |  |  |
| Smoking, n (%) |  |  |  |  |  |  |
| Never | 782 (55.8) | 78 (51.7) | 0.085 | NA | NA |  |
| Quit before pregnancy | 415 (29.6) | 58 (38.4) |  |  |  |  |
| Quit in first trimester of pregnancy | 139 (9.9) | 12 (7.9) |  |  |  |  |
| Continued smoking in pregnancy | 65 (4.6) | 3 (2.0) |  |  |  |  |
| *Missing* | *265* | *63* |  |  |  |  |
| Alcohol use, n (%) |  |  |  |  |  |  |
| No | 322 (21.1) | 30 (18.9) | 0.273 | NA | NA |  |
| <3 months before pregnancy | 932 (61.0) | 107 (67.3) |  |  |  |  |
| In pregnancy | 273 (17.9) | 22 (13.8) |  |  |  |  |
| *Missing* | *139* | *55* |  |  |  |  |
| Laboratory of chemicals measurements, n (%) |  |  |  |  |  |  |
| New York (NYU) | 1032 (61.9) | 149 (69.6) | 0.035 | 733 (65.1) | 57 (75.0) | 0.102 |
| Norway (NIPH) | 634 (38.1) | 65 (30.4) |  | 393 (34.9) | 19 (25.0) |  |
| *Missing* | *0* | *0* |  | *0* | *0* |  |
| Mode of conception, n (%) |  |  |  |  |  |  |
| Spontaneous conception | 1502 (90.2) | 189 (88.3) |  | 1029 (91.4) | 68 (89.5) |  |
| Assisted conception | 164 (9.8) | 25 (11.7) |  | 97 (8.6) | 8 (10.5) |  |
| No conception | 0 | 0 |  |  |  |  |
| Time to pregnancy, months, median (95% range) | 4.2 [0.0-78.5] | 6.6 [0.0-102.2] |  | 3.9 [0.0-64.5] | 5.3 [0.0-59.9] |  |
| *Missing* | *220* | *57* |  | *94* | *15* |  |
| Duration pursuing pregnancy in women that did not conceive, months, median (95% range) | NA | NA |  | NA | NA |  |
| *Missing* | *1666* | *214* |  | *1126* | *76* |  |
| Subfertility (time to pregnancy >12 months or assisted reproduction), n (%) |  |  |  |  |  |  |
| No | 1037 (71.2) | 93 (58.9) | 0.002 | 778 (75.2) | 43 (70.5) | 0.496 |
| Yes | 419 (28.8) | 65 (41.1) |  | 256 (24.8) | 18 (29.5) |  |
| *Missing* | *210* | *56* |  | *92* | *15* |  |
| Miscarriage, n (%) |  |  |  |  |  |  |
| No | 1666 (100.0) | 0 (0.0) | <0.001 | 1126 (100.0) | 0 (0.0) | <0.001 |
| Yes | 0 (0.0) | 214 (100.0) |  | 0 (0.0) | 76 (100.0) |  |
| *Missing* | *0* | *0* |  | *0* | *0* |  |
| History of miscarriage, n (%) |  |  |  |  |  |  |
| No | 1111 (80.7) | 114 (75.0) | 0.119 | NA | NA |  |
| Yes | 266 (19.3) | 38 (25.0) |  |  |  |  |
| *Missing* | *289* | *62* |  |  |  |  |
| Values presented as mean (SD), median (95% range) or number of participants (valid %)· NYU New York University NIPH Norwegian Institute of Public Health· | | | | | | |

| **Table S6.** Fecundability ratios for preconception individual and grouped urinary bisphenol and phthalate concentrations in women (N = 760), basic models. | | | |
| --- | --- | --- | --- |
| **Exposure** | **Fecundability Ratio (95% CI)** | **Nominal p-value** | **FDR p-value** |
| **Bisphenols** | | |  |
| Total bisphenols | 1·07 (0·95, 1·21) | 0·255 | 0·446 |
| BPA | 1·20 (1·06, 1·36) | 0·003* | 0·017* |
| BPS | 0·88 (0·79, 0·98) | 0·019* | 0·089 |
| **Phthalates** | | |  |
| PA | 1·06 (0·94, 1·18) | 0·336 | 0·523 |
| ***Low molecular weight (LMW) phthalates*** | 0·81 (0·72, 0·92) | 0·001* | 0·009* |
| mEP | 0·79 (0·70, 0·89) | <0·001* | 0·006* |
| mIBP | 0·94 (0·81, 1·08) | 0·377 | 0·556 |
| mBP | 0·91 (0·80, 1·04) | 0·174 | 0·375 |
| ***High molecular weight (HMW) phthalates*** |  |  |  |
| ***Di-2-ethylhexyl phthalate (DEHP)*** | 1·01 (0·89, 1·16) | 0·827 | 0·858 |
| mECPP | 1·02 (0·91, 1·15) | 0·717 | 0·834 |
| mCMHP | 1·11 (0·97, 1·28) | 0·131 | 0·331 |
| mEHHP | 1·01 (0·89, 1·14) | 0·923 | 0·923 |
| mEOHP | 1·03 (0·90, 1·17) | 0·678 | 0·825 |
| ***Di-n-octyl phthalate (DNOP)*** | 1·29 (1·03, 1·63) | 0·027* | 0·108 |
| mCPP | 1·11 (0·90, 1·36) | 0·315 | 0·519 |
| mCHpP | 1·31 (1·11, 1·54) | 0·001* | 0·009* |
| ***Diisononyl phthalate (DINP)*** |  |  |  |
| ohMiNP | 1·03 (0·84, 1·27) | 0·773 | 0·834 |
| oxoMiNP | 1·07 (0·83, 1·37) | 0·610 | 0·813 |
| cxMiNP/mCINP | 0·96 (0·89, 1·05) | 0·397 | 0·556 |
| mCIOP | 0·97 (0·85, 1·11) | 0·670 | 0·825 |
| ***Di(isononyl)cyclohexane-1,2-dicarboxylate (DINCH)*** |  |  |  |
| ohMINCH/mHNCH | 1·09 (0·97, 1·22) | 0·142 | 0·331 |
| oxoMINCH/mONCH | 1·14 (0·99, 1·32) | 0·072 | 0·240 |
| mCOCH | 0·92 (0·82, 1·02) | 0·119 | 0·331 |
| ***Di-propyl-heptyl phthalate (DiDP/DPHP)*** |  |  |  |
| oxomPHP | 1·02 (0·89, 1·16) | 0·774 | 0·834 |
| OHmPHP | 1·08 (0·96, 1·21) | 0·198 | 0·396 |
| cxmPHxP | 1·16 (0·98, 1·36) | 0·077 | 0·240 |
| ***Other*** |  |  |  |
| mHxP (DnHP) | 1·05 (0·97, 1·13) | 0·229 | 0·427 |
| mBzBP (BBzP) | 1·27 (1·10, 1·47) | 0·002* | 0·014* |
| Values represent change in fecundability per interquartile range increase in urinary exposure concentration. Cox proportional hazards models are adjusted for laboratory of analysis and urinary creatinine concentration. FDR: false discovery rate adjusted. *P-value <0·05. | | | |

| **Table S7.** Fecundability ratio for preconception individual and grouped urinary bisphenol and phthalate concentrations in women (N = 760), adjusted models**.** | | | |
| --- | --- | --- | --- |
| **Exposure** | **Fecundability Ratio (95% CI)** | **Nominal p-value** | **FDR p-value** |
| **Bisphenols** | | |  |
| Total bisphenols | 1·01 (0·89, 1·14) | 0·890 | 0·944 |
| BPA | 1·12 (0·99, 1·27) | 0·077 | 0·273 |
| BPS | 0·86 (0·77, 0·96) | 0·009* | 0·067 |
| **Phthalates** | | |  |
| PA | 1·01 (0·90, 1·13) | 0·910 | 0·944 |
| ***Low molecular weight (LMW) phthalates*** | 0·93 (0·81, 1·06) | 0·251 | 0·400 |
| mEP | 0·89 (0·78, 1·01) | 0·078 | 0·273 |
| mIBP | 1·06 (0·91, 1·22) | 0·477 | 0·668 |
| mBP | 1·00 (0·87, 1·15) | 0·985 | 0·985 |
| ***High molecular weight (HMW) phthalates*** |  |  |  |
| ***Di-2-ethylhexyl phthalate (DEHP)*** | 1·09 (0·96, 1·24) | 0·201 | 0·385 |
| mECPP | 1·09 (0·96, 1·23) | 0·180 | 0·385 |
| mCMHP | 1·20 (1·04, 1·38) | 0·010* | 0·067 |
| mEHHP | 1·08 (0·95, 1·22) | 0·257 | 0·400 |
| mEOHP | 1·09 (0·95, 1·25) | 0·206 | 0·384 |
| ***Di-n-octyl phthalate (DNOP)*** | 1·36 (1·07, 1·72) | 0·012* | 0·067 |
| mCPP | 1·17 (0·95, 1·45) | 0·144 | 0·367 |
| mCHpP | 1·34 (1·13, 1·58) | 0·001* | 0·022* |
| ***Diisononyl phthalate (DINP)*** |  |  |  |
| ohMiNP | 0·91 (0·72, 1·14) | 0·401 | 0·591 |
| oxoMiNP | 0·96 (0·73, 1·25) | 0·754 | 0·880 |
| cxMiNP/mCINP | 0·94 (0·85, 1·03) | 0·174 | 0·385 |
| mCIOP | 0·97 (0·84, 1·12) | 0·652 | 0·830 |
| ***Di(isononyl)cyclohexane-1,2-dicarboxylate (DINCH)*** |  |  |  |
| ohMINCH/mHNCH | 1·04 (0·92, 1·17) | 0·532 | 0·709 |
| oxoMINCH/mONCH | 1·09 (0·94, 1·27) | 0·236 | 0·400 |
| mCOCH | 0·88 (0·78, 0·99) | 0·028* | 0·131 |
| ***Di-propyl-heptyl phthalate (DiDP/DPHP)*** |  |  |  |
| oxomPHP | 1·03 (0·90, 1·17) | 0·703 | 0·856 |
| OHmPHP | 1·09 (0·97, 1·23) | 0·144 | 0·367 |
| cxmPHxP | 1·15 (0·97, 1·36) | 0·100 | 0·311 |
| ***Other*** |  |  |  |
| mHxP (DnHP) | 1·01 (0·93, 1·09) | 0·890 | 0·944 |
| mBzBP (BBzP) | 1·23 (1·06, 1·43) | 0·006* | 0·067 |
| Values represent change in fecundability per interquartile range increase in urinary exposure concentration. Cox proportional hazards models are adjusted for age, education, ethnicity, parity, smoking, alcohol use, folic acid supplement use, body mass index, laboratory of analysis, and maternal urinary creatinine concentration. FDR: false discovery rate adjusted. *P-value <0·s05. | | | |

| **Table S8.** Fecundability ratio for early pregnancy individual and grouped urinary bisphenol and phthalate concentrations in men (N=334), adjusted models**.** | | | |
| --- | --- | --- | --- |
| **Exposure** | **Fecundability Ratio (95% CI)** | **Nominal p-value** | **FDR p-value** |
| **Bisphenols** | | | |
| Total bisphenols | 1·07 (0·92, 1·26) | 0.385 | 0.898 |
| BPA | 1·17 (1·00, 1·38) | 0.056 | 0.308 |
| BPS | 0·87 (0·75, 1·00) | 0.049 | 0.308 |
| **Phthalates** | | | |
| PA | 1·15 (0·93, 1·42) | 0.183 | 0.641 |
| ***LMW phthalates*** | 1·11 (0·93, 1·32) | 0.236 | 0.720 |
| mEP | 1·17 (0·99, 1·39) | 0.066 | 0.308 |
| mIBP | 0·95 (0·82, 1·11) | 0.509 | 0.991 |
| mBP | 1·00 (0·87, 1·16) | 0.977 | 0.991 |
| ***HMW phthalates*** |  |  |  |
| ***DEHP*** | 1·00 (0·85, 1·18) | 0.973 | 0.991 |
| mECPP | 1·02 (0·85, 1·22) | 0.850 | 0.991 |
| mCMHP | 1·02 (0·87, 1·21) | 0.782 | 0.991 |
| mEHHP | 1·00 (0·86, 1·16) | 0.991 | 0.991 |
| mEOHP | 0·99 (0·85, 1·15) | 0.923 | 0.991 |
| ***DNOP*** | 1·11 (0·79, 1·56) | 0.537 | 0.991 |
| mCPP | 1·06 (0·75, 1·51) | 0.734 | 0.991 |
| mCHpP | 1·32 (1·09, 1·60) | 0.005* | 0.140 |
| ***DINP*** |  |  |  |
| ohMiNP | 1·77 (1·15, 2·76) | 0.011* | 0.154 |
| oxoMiNP | 1·71 (1·09, 2·69) | 0.020* | 0.187 |
| cxMiNP/mCINP | 1·06 (0·94, 1·19) | 0.326 | 0.830 |
| mCIOP | 0·97 (0·80, 1·17) | 0.728 | 0.991 |
| ***DINCH*** |  |  |  |
| ohMINCH/mHNCH | 1·01 (0·89, 1·16) | 0.856 | 0.991 |
| oxoMINCH/mONCH | 1·03 (0·86, 1·22) | 0.774 | 0.991 |
| mCOCH | 0·99 (0·84, 1·17) | 0.943 | 0.991 |
| ***DiDP*** |  |  |  |
| oxomPHP | 1·06 (0·89, 1·26) | 0.479 | 0.991 |
| OHmPHP | 1·11 (0·96, 1·27) | 0.152 | 0.608 |
| cxmPHxP | 1·07 (0·79, 1·45) | 0.664 | 0.991 |
| ***Other*** |  |  |  |
| mHxP (DnHP) | 0·90 (0·76, 1·08) | 0.257 | 0.720 |
| mBzBP (BBzP) | 1·00 (0·84, 1·21) | 0.959 | 0.991 |
| Values represent change in fecundability ratio per interquartile range increase in urinary exposure concentration. Cox proportional hazards models are adjusted for age, ethnicity, body mass index, laboratory of analysis and urinary creatinine concentration, and partners education, parity, smoking, alcohol use, and folic acid supplement use. FDR false discovery rate adjusted. *P-value <0·05. | | | |

| **Table S9.** Associations of preconception individual and grouped urinary bisphenol and phthalate concentrations with subfertility in women (N = 760), basic models. | | | |
| --- | --- | --- | --- |
| **Exposure** | **OR (95% CI)** | **Nominal p-value** | **FDR p-value** |
| **Bisphenols** | | |  |
| Total bisphenols | 0·86 (0·70, 1·06) | 0·165 | 0·308 |
| BPA | 0·73 (0·59, 0·91) | 0·004* | 0·037* |
| BPS | 1·10 (0·91, 1·32) | 0·335 | 0·494 |
| **Phthalates** | | |  |
| PA | 0·92 (0·75, 1·13) | 0·442 | 0·558 |
| ***Low molecular (LMW) weight phthalates*** | 1·26 (1·01, 1·58) | 0·038* | 0·140 |
| mEP | 1·37 (1·11, 1·7) | 0·004* | 0·037* |
| mIBP | 0·91 (0·7, 1·16) | 0·436 | 0·558 |
| mBP | 0·98 (0·77, 1·26) | 0·888 | 0·956 |
| ***High molecular weight (HMW) phthalates*** |  |  |  |
| ***Di-2-ethylhexyl phthalate (DEHP)*** | 0·81 (0·64, 1·02) | 0·072 | 0·183 |
| mECPP | 0·84 (0·68, 1·04) | 0·115 | 0·230 |
| mCMHP | 0·81 (0·64, 1·03) | 0·090 | 0·194 |
| mEHHP | 0·80 (0·64, 1·00) | 0·053 | 0·165 |
| mEOHP | 0·78 (0·61, 0·99) | 0·040* | 0·140 |
| ***Di-n-octyl phthalate (DNOP)*** | 0·57 (0·37, 0·86) | 0·008* | 0·042* |
| mCPP | 0·70 (0·48, 1·02) | 0·067 | 0·183 |
| mCHpP | 0·66 (0·49, 0·89) | 0·006* | 0·042* |
| ***Diisononyl phthalate (DINP)*** |  |  |  |
| ohMiNP | 1·00 (0·70, 1·43) | 0·992 | 0·992 |
| oxoMiNP | 0·96 (0·62, 1·48) | 0·858 | 0·956 |
| cxMiNP/mCINP | 1·06 (0·91, 1·23) | 0·458 | 0·558 |
| mCIOP | 0·99 (0·78, 1·26) | 0·935 | 0·970 |
| ***Di(isononyl)cyclohexane-1,2-dicarboxylate (DINCH)*** |  |  |  |
| ohMINCH/mHNCH | 0·89 (0·73, 1·10) | 0·283 | 0·466 |
| oxoMINCH/mONCH | 0·86 (0·68, 1·09) | 0·207 | 0·363 |
| mCOCH | 1·29 (1·07, 1·57) | 0·009* | 0·042* |
| ***Di-propyl-heptyl phthalate (DiDP/DPHP)*** |  |  |  |
| oxomPHP | 0·91 (0·73, 1·14) | 0·427 | 0·558 |
| OHmPHP | 0·81 (0·63, 1·03) | 0·085 | 0·194 |
| cxmPHxP | 0·86 (0·63, 1·16) | 0·326 | 0·494 |
| ***Other*** |  |  |  |
| mHxP (DnHP) | 0·96 (0·83, 1·10) | 0·553 | 0·645 |
| mBzBP (BBzP) | 0·61 (0·47, 0·80) | <0·001* | 0·006* |
| Values represent odds of subfertility (odds ratio (OR), 95% confidence interval (CI)) per interquartile range increase in urinary exposure concentration. Logistic regression models are adjusted for laboratory of analysis and urinary creatinine concentration. FDR: false discovery rate adjusted. *P-value <0·05. | | | |

| **Table S10.** Associations of preconception individual and grouped urinary bisphenol and phthalate concentrations with subfertility in women (N = 760), adjusted models. | | | |
| --- | --- | --- | --- |
| **Exposure** | **OR (95% CI)** | **Nominal p-value** | **FDR p-value** |
| **Bisphenols** | | |  |
| Total bisphenols | 1·01 (0·80, 1·27) | 0·956 | 0·990 |
| BPA | 0·85 (0·68, 1·07) | 0·162 | 0·349 |
| BPS | 1·12 (0·91, 1·38) | 0·274 | 0·488 |
| **Phthalates** | | |  |
| PA | 0·94 (0·75, 1·18) | 0·599 | 0·735 |
| ***Low molecular weight (LMW) phthalates*** | 1·07 (0·83, 1·37) | 0·607 | 0·735 |
| mEP | 1·18 (0·93, 1·50) | 0·179 | 0·358 |
| mIBP | 0·79 (0·59, 1·04) | 0·093 | 0·237 |
| mBP | 0·87 (0·66, 1·15) | 0·335 | 0·521 |
| ***High molecular weight (HMW) phthalates*** |  |  |  |
| ***Di-2-ethylhexyl phthalate (DEHP)*** | 0·78 (0·61, 1·01) | 0·059 | 0·207 |
| mECPP | 0·82 (0·64, 1·03) | 0·092 | 0·237 |
| mCMHP | 0·78 (0·60, 1·01) | 0·056 | 0·207 |
| mEHHP | 0·77 (0·60, 0·99) | 0·040* | 0·207 |
| mEOHP | 0·77 (0·59, 1·00) | 0·046* | 0·207 |
| ***Di-n-octyl phthalate (DNOP)*** | 0·55 (0·35, 0·87) | 0·010* | 0·070 |
| mCPP | 0·73 (0·48, 1·10) | 0·133 | 0·310 |
| mCHpP | 0·59 (0·42, 0·83) | 0·002* | 0·028* |
| ***Diisononyl phthalate (DINP)*** |  |  |  |
| ohMiNP | 1·10 (0·72, 1·67) | 0·669 | 0·749 |
| oxoMiNP | 0·99 (0·60, 1·64) | 0·990 | 0·990 |
| cxMiNP/mCINP | 1·09 (0·92, 1·29) | 0·299 | 0·492 |
| mCIOP | 0·97 (0·75, 1·26) | 0·834 | 0·898 |
| ***Di(isononyl)cyclohexane-1,2-dicarboxylate (DINCH)*** |  |  |  |
| ohMINCH/mHNCH | 0·92 (0·74, 1·15) | 0·481 | 0·673 |
| oxoMINCH/mONCH | 0·89 (0·68, 1·17) | 0·401 | 0·591 |
| mCOCH | 1·34 (1·08, 1·65) | 0·007* | 0·065 |
| ***Di-propyl-heptyl phthalate (DiDP/DPHP)*** |  |  |  |
| oxomPHP | 0·94 (0·74, 1·19) | 0·590 | 0·735 |
| OHmPHP | 0·79 (0·61, 1·03) | 0·078 | 0·237 |
| cxmPHxP | 0·84 (0·60, 1·16) | 0·279 | 0·488 |
| ***Other*** |  |  |  |
| mHxP (DnHP) | 1·04 (0·89, 1·22) | 0·630 | 0·735 |
| mBzBP (BBzP) | 0·59 (0·44, 0·79) | 0·001* | 0·028* |
| Values represent odds of subfertility (odds ratio (OR), 95% confidence interval (CI)) per interquartile range increase in urinary exposure concentration. Logistic regression models are adjusted for age, education, ethnicity, parity, smoking, alcohol use, folic acid supplement use, body mass index, laboratory of analysis, and maternal urinary creatinine concentration. FDR: false discovery rate adjusted. *P-value <0·05. | | | |

| **Table S11.** Associations of early pregnancy individual and grouped urinary bisphenol and phthalate concentrations with subfertility in men (N=334), adjusted models. | | | |
| --- | --- | --- | --- |
| **Exposure** | **OR (95% CI)** | **Nominal p-value** | **FDR p-value** |
| **Bisphenols** | | | |
| Total bisphenols | 0·91 (0·64, 1·30) | 0.609 | 0.991 |
| BPA | 0·73 (0·51, 1·04) | 0.099 | 0.495 |
| BPS | 1·37 (1·00, 0·88) | 0.048* | 0.457 |
| **Phthalates** | | | |
| PA | 0·74 (0·47, 1·17) | 0.197 | 0.788 |
| ***LMW phthalates*** | 0·70 (0·49, 1·00) | 0.049* | 0.457 |
| mEP | 0·63 (0·43, 0·91) | 0.014* | 0.392 |
| mIBP | 1·02 (0·73, 1·42) | 0.908 | 0.991 |
| mBP | 0·92 (0·66, 1·27) | 0.608 | 0.991 |
| ***HMW phthalates*** |  |  |  |
| ***DEHP*** | 0·95 (0·65, 1·37) | 0.770 | 0.991 |
| mECPP | 0·89 (0·59, 1·33) | 0.572 | 0.991 |
| mCMHP | 0·97 (0·68, 1·40) | 0.889 | 0.991 |
| mEHHP | 0·97 (0·68, 1·39) | 0.877 | 0.991 |
| mEOHP | 0·96 (0·67, 1·37) | 0.819 | 0.991 |
| ***DNOP*** | 0·83 (0·41, 1·67) | 0.595 | 0.991 |
| mCPP | 0·94 (0·45, 1·97) | 0.866 | 0.991 |
| mCHpP | 1·00 (1·00, 1·00) | 0.991 | 0.991 |
| ***DINP*** |  |  |  |
| ohMiNP | 0·49 (0·20, 1·17) | 0.106 | 0.495 |
| oxoMiNP | 0·42 (0·17, 1·07) | 0.067 | 0.469 |
| cxMiNP/mCINP | 0·98 (0·82, 1·16) | 0.800 | 0.991 |
| mCIOP | 0·99 (0·63, 1·53) | 0.948 | 0.991 |
| ***DINCH*** |  |  |  |
| ohMINCH/mHNCH | 0·91 (0·67, 1·24) | 0.566 | 0.991 |
| oxoMINCH/mONCH | 0·96 (0·63, 1·45) | 0.847 | 0.991 |
| mCOCH | 1·08 (0·71, 1·63) | 0.729 | 0.991 |
| ***DiDP/DPHP*** |  |  |  |
| oxomPHP | 1·10 (0·75, 1·61) | 0.640 | 0.991 |
| OHmPHP | 0·95 (0·70, 1·28) | 0.737 | 0.991 |
| cxmPHxP | 1·01 (0·49, 2·08) | 0.969 | 0.991 |
| ***Other*** |  |  |  |
| mHxP (DnHP) | 1·24 (0·86, 1·79) | 0.253 | 0.886 |
| mBzBP (BBzP) | 0·80 (0·53, 1·22) | 0.304 | 0.946 |
| Value represent odds on subfertility (odds ratio (OR), 95% confidence interval (CI)) per interquartile range increase in urinary exposure concentrations. Logistic regression models are adjusted for age, ethnicity, body mass index, laboratory of analysis and urinary creatinine concentration, and partners education, parity, smoking, alcohol use, and folic acid supplement use. FDR false discovery rate adjusted. *P-value <0·05. | | | |

| **Table S12.** Associations of periconception individual and grouped urinary bisphenol and phthalate concentrations with miscarriage in women (N = 1880), basic models. | | | |
| --- | --- | --- | --- |
| **Exposure** | **OR (95% CI)** | **P-value** | **FDR p-value** |
| **Bisphenols** | | |  |
| Total bisphenols | 0·80 (0·65, 1·00) | 0.045 | 0.158 |
| BPA | 0·82 (0·65, 1·04) | 0.107 | 0.311 |
| BPS | 0·79 (0·64, 0·97) | 0.024* | 0.134 |
| **Phthalates** | | |  |
| PA | 0·77 (0·64, 0·94) | 0.008* | 0.075 |
| ***Low molecular weight (LMW) phthalates*** | 0·91 (0·74, 1·14) | 0.417 | 0.649 |
| mEP | 0·94 (0·77, 1·16) | 0.587 | 0.783 |
| mIBP | 1 ·00 (0·78, 1·27) | 0.987 | 0.987 |
| mBP | 0·88 (0·69, 1·13) | 0.316 | 0.553 |
| ***High molecular weight (HMW) phthalates*** |  |  |  |
| ***Di-2-ethylhexyl phthalate (DEHP)*** | 0·97 (0·77, 1·22) | 0.799 | 0.973 |
| mECPP | 0·91 (0·73, 1·13) | 0.385 | 0.634 |
| mCMHP | 1·16 (0·96, 1·41) | 0.122 | 0.311 |
| mEHHP | 1·01 (0·81, 1·26) | 0.939 | 0.980 |
| mEOHP | 0·92 (0·74, 1·16) | 0.499 | 0.699 |
| ***Di-n-octyl phthalate (DNOP)*** | 0·65 (0·44, 0·97) | 0.034* | 0.158 |
| mCPP | 1·01 (0·71, 1·45) | 0.945 | 0.980 |
| mCHpP | 0·62 (0·45, 0·85) | 0.003* | 0.042* |
| ***Diisononyl phthalate (DINP)*** |  |  |  |
| ohMiNP | 0·74 (0·47, 1·15) | 0.177 | 0.364 |
| oxoMiNP | 0·65 (0·37, 1·12) | 0.119 | 0.311 |
| cxMiNP/mCINP | 0·91 (0·78, 1·05) | 0.195 | 0.364 |
| mCIOP | 1·26 (1·01, 1·58) | 0.045* | 0.158 |
| ***Di(isononyl)cyclohexane-1,2-dicarboxylate (DINCH)*** |  |  |  |
| ohMINCH/mHNCH | 1·01 (0·84, 1·23) | 0.902 | 0.980 |
| oxoMINCH/mONCH | 0·97 (0·77, 1·22) | 0.786 | 0.973 |
| mCOCH | 1·27 (1·06, 1·53) | 0.011* | 0.077 |
| ***Di-propyl-heptyl phthalate (DiDP/DPHP)*** |  |  |  |
| oxomPHP | 1·18 (0·95, 1·47) | 0.137 | 0.120 |
| OHmPHP | 0·92 (0·73, 1·15) | 0.450 | 0.663 |
| cxmPHxP | 1·83 (1·39, 2·40) | <0.001* | 0.006* |
| ***Other*** |  |  |  |
| mHxP (DnHP) | 0·99 (0·86, 1·15) | 0.932 | 0.980 |
| mBzBP (BBzP) | 0·84 (0·65, 1·09) | 0.195 | 0.364 |
| Values represent odds of miscarriage (odds ratio (OR), 95% confidence interval (CI)) per interquartile range increase in urinary exposure concentration. Logistic regression models are adjusted for laboratory of analysis and urinary creatinine concentration. FDR: false discovery rate adjusted. *P-value <0·05. | | | |

| **Table S13.** Associations of periconception individual and grouped urinary bisphenol and phthalate concentrations with miscarriage in women (N = 1880), adjusted models. | | | |
| --- | --- | --- | --- |
| **Exposure** | **OR (95% CI)** | **Nominal p-value** | **FDR p-value** |
| **Bisphenols** | | |  |
| Total bisphenols | 0·81 (0·65, 1·01) | 0.057 | 0.200 |
| BPA | 0·83 (0·65, 1·06) | 0.135 | 0.341 |
| BPS | 0·79 (0·64, 0·97) | 0.024* | 0.112 |
| **Phthalates** | | |  |
| PA | 0·79 (0·65, 0·96) | 0.019* | 0.112 |
| ***Low molecular weight (LMW) phthalates*** | 0·89 (0·71, 1·11) | 0.306 | 0.504 |
| mEP | 0·92 (0·75, 1·15) | 0.478 | 0.661 |
| mIBP | 0·98 (0·77, 1·25) | 0.861 | 0.952 |
| mBP | 0·87 (0·68, 1·12) | 0.271 | 0.474 |
| ***High molecular weight (HMW) phthalates*** |  |  |  |
| ***Di-2-ethylhexyl phthalate (DEHP)*** | 0·96 (0·77, 1·21) | 0.754 | 0.952 |
| mECPP | 0·90 (0·72, 1·12) | 0.351 | 0.517 |
| mCMHP | 1·16 (0·95, 1·41) | 0.146 | 0.341 |
| mEHHP | 1·00 (0·80, 1·25) | 0.980 | 0.980 |
| mEOHP | 0·92 (0·73, 1·16) | 0.496 | 0.661 |
| ***Di-n-octyl phthalate (DNOP)*** | 0·62 (0·41, 0·93) | 0.021 | 0.112 |
| mCPP | 0·98 (0·68, 1·42) | 0.933 | 0.968 |
| mCHpP | 0·59 (0·43, 0·82) | 0.002* | 0.028* |
| ***Diisononyl phthalate (DINP)*** |  |  |  |
| ohMiNP | 0·74 (0·47, 1·16) | 0.191 | 0.357 |
| oxoMiNP | 0·64 (0·37, 1·11) | 0.112 | 0.341 |
| cxMiNP/mCINP | 0·90 (0·77, 1·05) | 0.170 | 0.348 |
| mCIOP | 1·29 (1·02, 1·63) | 0.030* | 0.120 |
| ***Di(isononyl)cyclohexane-1,2-dicarboxylate (DINCH)*** |  |  |  |
| ohMINCH/mHNCH | 1·02 (0·84, 1·24) | 0.860 | 0.952 |
| oxoMINCH/mONCH | 0·97 (0·77, 1·23) | 0.815 | 0.952 |
| mCOCH | 1·30 (1·08, 1·58) | 0.006* | 0.056 |
| ***Di-propyl-heptyl phthalate (DiDP/DPHP)*** |  |  |  |
| oxomPHP | 1·19 (0·95, 1·48) | 0.136 | 0.341 |
| OHmPHP | 0·89 (0·71, 1·13) | 0.344 | 0.517 |
| cxmPHxP | 1·89 (1·43, 2·51) | <0.001* | 0.006* |
| ***Other*** |  |  |  |
| mHxP (DnHP) | 1·01 (0·87, 1·18) | 0.884 | 0.952 |
| mBzBP (BBzP) | 0·83 (0·64, 1·09) | 0.174 | 0.348 |
| Values represent odds of miscarriage (odds ratio (OR), 95% confidence interval (CI)) per interquartile range increase in urinary exposure concentration. Logistic regression models are adjusted for age, education, ethnicity, parity, smoking, alcohol use, folic acid supplement use, body mass index, laboratory of analysis, and urinary creatinine concentration. FDR false discovery rate adjusted. *P-value <0·05. | | | |

| **Table S14.** Associations of early pregnancy individual and grouped urinary bisphenol and phthalate concentrations with miscarriage in men (N =1202), adjusted models. | | | |
| --- | --- | --- | --- |
| **Exposure** | **OR (95% CI)** | **Nominal p-value** | **FDR p-value** |
| **Bisphenols** | | |  |
| Total bisphenols | 1·03 (0·73, 1·44) | 0·878 | 0·941 |
| BPA | 1·10 (0·78, 1·55) | 0·594 | 0·767 |
| BPS | 1·12 (0·85, 1·46) | 0·422 | 0·767 |
| **Phthalates** | | |  |
| PA | 0·95 (0·66, 1·37) | 0·768 | 0·860 |
| ***LMW phthalates*** | 1·09 (0·76, 1·57) | 0·628 | 0·767 |
| mEP | 1·06 (0·74, 1·51) | 0·762 | 0·860 |
| mIBP | 1·00 (0·70, 1·44) | 0·981 | 0·981 |
| mBP | 1·11 (0·80, 1·52) | 0·533 | 0·767 |
| ***HMW phthalates*** |  |  |  |
| ***DEHP*** | 1·20 (0·84, 1·71) | 0·310 | 0·767 |
| mECPP | 1·05 (0·72, 1·52) | 0·809 | 0·767 |
| mCMHP | 0·90 (0·66, 1·24) | 0·523 | 0·767 |
| mEHHP | 1·23 (0·87, 1·75) | 0·242 | 0·767 |
| mEOHP | 1·23 (0·87, 1·73) | 0·243 | 0·767 |
| ***DNOP*** | 1·68 (0·92, 3·08) | 0·090 | 0·767 |
| mCPP | 1·17 (0·62, 2·21) | 0·630 | 0·767 |
| mCHpP | 1·40 (1·11, 1·76) | 0·005* | 0·140 |
| ***DINP*** |  |  |  |
| ohMiNP | 0·64 (0·29, 1·46) | 0·289 | 0·767 |
| oxoMiNP | 0·63 (0·27, 1·47) | 0·287 | 0·767 |
| cxMiNP/mCINP | 0·94 (0·79, 1·12) | 0·470 | 0·767 |
| mCIOP | 1·10 (0·78, 1·54) | 0·602 | 0·767 |
| ***DINCH*** |  |  |  |
| ohMINCH/mHNCH | 1·25 (0·92, 1·69) | 0·150 | 0·767 |
| oxoMINCH/mONCH | 1·17 (0·83, 1·66) | 0·361 | 0·767 |
| mCOCH | 1·32 (0·94, 1·864 | 0·110 | 0·767 |
| ***DiDP/DPHP*** |  |  |  |
| oxomPHP | 1·31 (0·92, 1·85) | 0·129 | 0·767 |
| OHmPHP | 0·91 (0·68, 1·23) | 0·551 | 0·767 |
| cxmPHxP | 0·96 (0·52, 1·77) | 0·907 | 0·941 |
| ***Other*** |  |  |  |
| mHxP (DnHP) | 0·85 (0·58, 1·25) | 0·405 | 0·767 |
| mBzBP (BBzP) | 1·11 (0·73, 1·71) | 0·620 | 0·767 |
| Values represent odds of miscarriage (odds ratio (OR), 95% confidence interval (CI)) per interquartile range increase in urinary exposure concentration. Logistic regression models are adjusted for age, ethnicity, body mass index, laboratory of analysis and urinary creatinine concentration, and partners education, parity, smoking, alcohol use, and folic acid supplement use. FDR false discovery rate adjusted. *P-value <0·05. | | | |

| **Table S15.** Fecundability ratios for preconception individual and grouped urinary bisphenols and phthalate metabolites in women who conceived naturally (N = 652), adjusted models**.** | | | |
| --- | --- | --- | --- |
| **Exposure** | **Fecundability Ratio (95% CI)** | **Nominal p-value** | **FDR p-value** |
| **Bisphenols** | | |  |
| Total bisphenols | 1·05 (0·91, 1·20) | 0·496 | 0·694 |
| BPA | 1·19 (1·02, 1·38) | 0·024* | 0·168 |
| BPS | 0·87 (0·77, 0·98) | 0·024* | 0·168 |
| **Phthalates** | | |  |
| PA | 1·00 (0·89, 1·14) | 0·942 | 0·977 |
| ***Low molecular weight (LMW) phthalates*** | 0·90 (0·78, 1·04) | 0·149 | 0·417 |
| mEP | 0·87 (0·75, 1·00) | 0·047* | 0·188 |
| mIBP | 1·07 (0·90, 1·26) | 0·448 | 0·694 |
| mBP | 1·00 (0·85, 1·17) | 0·992 | 0·992 |
| ***High molecular weight (HMW) phthalates*** |  |  |  |
| ***Di-2-ethylhexyl phthalate (DEHP)*** | 1·05 (0·92, 1·21) | 0·473 | 0·694 |
| mECPP | 1·06 (0·93, 1·20) | 0·369 | 0·689 |
| mCMHP | 1·18 (1·01, 1·37) | 0·038* | 0·182 |
| mEHHP | 1·04 (0·91, 1·20) | 0·553 | 0·704 |
| mEOHP | 1·06 (0·92, 1·23) | 0·415 | 0·694 |
| ***Di-n-octyl phthalate (DNOP)*** | 1·31 (1·01, 1·70) | 0·039* | 0·182 |
| mCPP | 1·14 (0·90, 1·44) | 0·280 | 0·560 |
| mCHpP | 1·31 (1·10, 1·57) | 0·003* | 0·042* |
| ***Diisononyl phthalate (DINP)*** |  |  |  |
| ohMiNP | 0·96 (0·76, 1·21) | 0·727 | 0·874 |
| oxoMiNP | 1·03 (0·78, 1·36) | 0·831 | 0·931 |
| cxMiNP/mCINP | 0·93 (0·84, 1·02) | 0·133 | 0·414 |
| mCIOP | 0·94 (0·81, 1·10) | 0·456 | 0·694 |
| ***Di(isononyl)cyclohexane-1,2-dicarboxylate (DINCH)*** |  |  |  |
| ohMINCH/mHNCH | 1·04 (0·91, 1·19) | 0·541 | 0·704 |
| oxoMINCH/mONCH | 1·11 (0·94, 1·30) | 0·212 | 0·457 |
| mCOCH | 0·89 (0·79, 1·01) | 0·069 | 0·242 |
| ***Di-propyl-heptyl phthalate (DiDP/DPHP)*** |  |  |  |
| oxomPHP | 0·99 (0·86, 1·14) | 0·868 | 0·935 |
| OHmPHP | 1·09 (0·96, 1·22) | 0·185 | 0·432 |
| cxmPHxP | 1·14 (0·94, 1·37) | 0·175 | 0·432 |
| ***Other*** |  |  |  |
| mHxP (DnHP) | 0·99 (0·90, 1·08) | 0·749 | 0·874 |
| mBzBP (BBzP) | 1·31 (1·10, 1·55) | 0·002* | 0·042* |
| Values represent change in fecundability ratio per interquartile range increase in urinary exposure concentration. Cox proportional hazards models are adjusted for age, education, ethnicity, parity, smoking, alcohol use, folic acid supplement use, body mass index, laboratory of analysis, and maternal urinary creatinine concentration. FDR: false discovery rate adjusted. *P-value <0·05. | | | |

| **Table S16.** Associations of preconception individual and grouped urinary bisphenol and phthalate concentrations with subfertility in women who conceived naturally(N=621), adjusted models. | | | |
| --- | --- | --- | --- |
| **Exposure** | **OR(95% CI)** | **Nominal p-value** | **FDR p-value** |
| **Bisphenols** | | |  |
| Total bisphenols | 0·88 (0·68, 1·15) | 0·360 | 0·465 |
| BPA | 0·72 (0·55, 0·94) | 0·017 | 0·119 |
| BPS | 1·14 (0·91, 1·43) | 0·259 | 0·465 |
| **Phthalates** | | |  |
| PA | 0·88 (0·68, 1·14) | 0·328 | 0·465 |
| ***Low molecular weight(LMW) phthalates*** | 1·14 (0·86, 1·51) | 0·365 | 0·465 |
| mEP | 1·26 (0·96, 1·65) | 0·094 | 0·306 |
| mIBP | 0·77 (0·56, 1·06) | 0·108 | 0·306 |
| mBP | 0·86 (0·64, 1·17) | 0·348 | 0·465 |
| ***High molecular weight(HMW) phthalates*** |  |  |  |
| ***Di-2-ethylhexyl phthalate(DEHP)*** | 0·80 (0·60, 1·07) | 0·131 | 0·306 |
| mECPP | 0·83 (0·64, 1·08) | 0·174 | 0·364 |
| mCMHP | 0·80 (0·60, 1·07) | 0·131 | 0·306 |
| mEHHP | 0·80 (0·61, 1·05) | 0·114 | 0·306 |
| mEOHP | 0·79 (0·58, 1·06) | 0·112 | 0·306 |
| ***Di-n-octyl phthalate(DNOP)*** | 0·58 (0·35, 0·96) | 0·033 | 0·185 |
| mCPP | 0·78 (0·50, 1·24) | 0·293 | 0·465 |
| mCHpP | 0·56 (0·38, 0·83) | 0·004* | 0·056 |
| ***Diisononyl phthalate(DINP)*** |  |  |  |
| ohMiNP | 1·02 (0·63, 1·64) | 0·945 | 0·961 |
| oxoMiNP | 0·86 (0·48, 1·55) | 0·617 | 0·691 |
| cxMiNP/mCINP | 1·11 (0·92, 1·34) | 0·270 | 0·465 |
| mCIOP | 1·01 (0·76, 1·34) | 0·961 | 0·961 |
| ***Di(isononyl)cyclohexane-1.2-dicarboxylate(DINCH)*** |  |  |  |
| ohMINCH/mHNCH | 0·93 (0·73, 1·19) | 0·571 | 0·666 |
| oxoMINCH/mONCH | 0·87 (0·64, 1·19) | 0·394 | 0·480 |
| mCOCH | 1·36 (1·07, 1·73) | 0·011* | 0·103 |
| ***Di-propyl-heptyl phthalate(DiDP/DPHP)*** |  |  |  |
| oxomPHP | 1·02 (0·79, 1·33) | 0·875 | 0·942 |
| OHmPHP | 0·81 (0·63, 1·04) | 0·094 | 0·306 |
| cxmPHxP | 0·78 (0·54, 1·13) | 0·182 | 0·364 |
| ***Other*** |  |  |  |
| mHxP(DnHP) | 1·09 (0·92, 1·30) | 0·317 | 0·465 |
| mBzBP(BBzP) | 0·53 (0·38, 0·75) | <0·001* | 0·006* |
| Values represent odds of subfertility(odds ratio(OR). 95% confidence interval(CI)) per interquartile range increase in urinary exposure concentration. Logistic regression models are adjusted for age, education, ethnicity, parity, smoking, alcohol use, folic acid supplement use, body mass index, laboratory of analysis, and maternal urinary creatinine concentration. FDR: false discovery rate adjusted. *P-value <0·05. | | | |

| **Table S17.** Associations of periconception individual and grouped urinary bisphenol and phthalate concentrations with miscarriage in women who conceived naturally (N=1691), adjusted models. | | | |
| --- | --- | --- | --- |
| **Exposure** | **OR (95% CI)** | **Nominal p-value** | **FDR p-value** |
| **Bisphenols** | | |  |
| Total bisphenols | 0·83 (0·56, 1·21) | 0.331 | 0.729 |
| BPA | 0·87 (0·59, 1·29) | 0.495 | 0.729 |
| BPS | 0·80 (0·55, 1·16) | 0.239 | 0.729 |
| **Phthalates** | | |  |
| PA | 0·88 (0·62, 1·24) | 0.465 | 0.729 |
| ***Low molecular weight (LMW) phthalates*** | 0·87 (0·57, 1·34) | 0.536 | 0.750 |
| mEP | 0·95 (0·64, 1·41) | 0.791 | 0.820 |
| mIBP | 0·78 (0·50, 1·21) | 0.272 | 0.729 |
| mBP | 0·83 (0·54, 1·28) | 0.400 | 0.729 |
| ***High molecular weight (HMW) phthalates*** |  |  |  |
| ***Di-2-ethylhexyl phthalate (DEHP)*** | 0·93 (0·62, 1·4) | 0.732 | 0.788 |
| mECPP | 0·85 (0·58, 1·24) | 0.397 | 0.729 |
| mCMHP | 1·16 (0·79, 1·69) | 0.444 | 0.729 |
| mEHHP | 0·96 (0·64, 1·43) | 0.824 | 0.824 |
| mEOHP | 0·91 (0·59, 1·39) | 0.656 | 0.765 |
| ***Di-n-octyl phthalate (DNOP)*** | 0·63 (0·31, 1·32) | 0.221 | 0.729 |
| mCPP | 0·76 (0·40, 1·43) | 0.388 | 0.729 |
| mCHpP | 0·88 (0·53, 1·47) | 0.636 | 0.765 |
| ***Diisononyl phthalate (DINP)*** |  |  |  |
| ohMiNP | 0·55 (0·22, 1·35) | 0.187 | 0.729 |
| oxoMiNP | 0·56 (0·19, 1·64) | 0.286 | 0.729 |
| cxMiNP/mCINP | 0·89 (0·67, 1·19) | 0.442 | 0.729 |
| mCIOP | 0·90 (0·62, 1·32) | 0.594 | 0.756 |
| ***Di(isononyl)cyclohexane-1,2-dicarboxylate (DINCH)*** |  |  |  |
| ohMINCH/mHNCH | 1·16 (0·82, 1·64) | 0.415 | 0.729 |
| oxoMINCH/mONCH | 1·08 (0·72, 1·61) | 0.717 | 0.788 |
| mCOCH | 1·19 (0·87, 1·62) | 0.284 | 0.729 |
| ***Di-propyl-heptyl phthalate (DiDP/DPHP)*** |  |  |  |
| oxomPHP | 0·91 (0·65, 1·28) | 0.594 | 0.756 |
| OHmPHP | 0·87 (0·59, 1·26) | 0.454 | 0.729 |
| cxmPHxP | 1·20 (0·73, 1·97) | 0.479 | 0.729 |
| ***Other*** |  |  |  |
| mHxP (DnHP) | 0·84 (0·64, 1·11) | 0.216 | 0.729 |
| mBzBP (BBzP) | 0·77 (0·48, 1·23) | 0.270 | 0.729 |
| Values represent odds of subfertility (odds ratio (OR), 95% confidence interval (CI)) per interquartile range increase in urinary exposure concentration. Logistic regression models are adjusted for age, education, ethnicity, parity, smoking, alcohol use, folic acid supplement use, body mass index, laboratory of analysis, and maternal urinary creatinine concentration. FDR: false discovery rate adjusted. | | | |

**Figure S1.** Directed Acyclic Graph

**Figure S2.** Correlations of urinary bisphenol and phthalate metabolite concentrations. A) Women, measured in New York University (NYU) laboratory, B) women measured in Norwegian Institute of Public Health (NIPH) laboratory, C) men, measured in NYU laboratory, D) men, measured in NIPH laboratory.
